# L1CAM promotes vasculogenic mimicry formation by miR‐143‐3p‐induced expression of hexokinase 2 in glioma

**DOI:** 10.1002/1878-0261.13384

**Published:** 2023-02-08

**Authors:** Yishan Huang, Chenchen Zhu, Pei Liu, Fan Ouyang, Juanjuan Luo, Chunjiao Lu, Bo Tang, Xiaojun Yang

**Affiliations:** ^1^ Guangdong Provincial Key Laboratory of Infectious Disease and Molecular Immunopathology Shantou University Medical College China; ^2^ Department of Hepatobiliary Surgery The First Affiliated Hospital of Guangxi Medical University Nanning China

**Keywords:** glioma, HK2, L1CAM, miR‐143‐3p, vasculogenic mimicry

## Abstract

In recent decades, antiangiogenic therapy, which blocks the supply of oxygen and nutrition to tumor cells, has become a promising clinical strategy for the treatment of patients with tumors. However, recent studies revealed that vasculogenic mimicry (VM), which is the process by which vascular morphological structures are formed by highly invasive tumor cells, has been considered a potential factor for the failure of antiangiogenic therapy in patients with tumors. Thus, inhibition of VM formation might be a potential target for improving the outcome of antiangiogenic strategies. However, the mechanism underlying VM formation is still incompletely elucidated. Herein, we report that L1CAM might be a critical regulator of VM formation in glioma, and might be associated with the resistance of glioma to antiangiogenic therapy. We found that the tumor‐invasion and tube‐formation capabilities of *L1CAM*‐overexpressing cells were significantly enhanced *in vitro* and *in vivo*. In addition, the results indicated that miR‐143‐3p, which might directly target the 3'UTR of the hexokinase 2 (*HK2*) gene to regulate its protein expression, was subsequently involved in L1CAM‐mediated VM formation by glioma cells. Further study revealed that the regulation of *MMP2*, *MMP9,* and *VEGFA* expression was involved in this process. Moreover, we identified that activation of the downstream PI3K/AKT signaling pathway of the L1CAM/HK2 cascade is critical for VM formation by glioma cells. Furthermore, we found that the combined treatment of anti‐L1CAM neutralizing monoclonal antibody and bevacizumab increases efficacy beyond that of bevacizumab alone, and suppresses glioma growth *in vivo*, indicating that the inhibition of L1CAM‐mediated VM formation might efficiently improve the effect of antiangiogenic treatment for glioma patients. Together, our findings demonstrated a critical role of L1CAM in regulating VM formation in glioma, and that L1CAM might be a potential target for ameliorating tumor resistance to antiangiogenic therapy in glioma patients.

## Graphical abstract

The authors identified L1CAM as a crucial regulatory factor for modulating vasculogenic mimicry formation in glioma, which might be a potential target for ameliorating tumor resistance to antiangiogenic therapy in glioma patients.

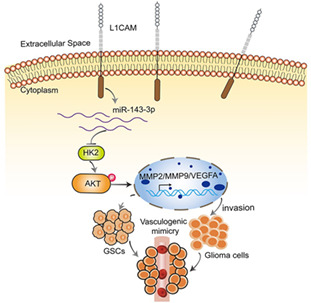

**Abbreviations**
bFGF: basic fibroblast growth factor
CNS: central nervous system
CSCs: cancer stem cells
DEGs: differently expressed genes
EGCG: epigallocatechin‐3‐gallate
EGF: epidermal Growth Factor
EMT: epithelial‐mesenchymal transition
EphA2: EPH receptor A2
FGF1: fibroblast growth factor 1
GBM: glioblastoma
GO: gene ontology
GSCs: glioma stem cells
HK2: hexokinase 2
IHC: immunohistochemistry
L1CAM/L1: neural cell adhesion molecule L1
miRNA: microRNA
MMP2: matrix metalloproteinase 2
MMP9: matrix metalloproteinase 9
MMPs: matrix metalloproteinases
PI3K: phosphatidylinositol 3‐kinase
qRT‐PCR: quantitative real‐time polymerase chain reaction
siRNA: small‐interfering RNA
TNBC: triple‐negative breast cancer
VE‐cadherin: vascular endothelial cadherin
VEGFA: vascular endothelial growth factor A
VEGFR: vascular endothelial growth factor receptor
VM: vasculogenic mimicry

## 1 Introduction

Glioma, which accounts for more than 75% of brain tumors, is the most common tumor in the central nervous system [1]. Among gliomas, glioblastoma (GBM) is one of the most lethal, with an average survival time of only ~ 14.6 months even after complete surgical resection combined with standard chemotherapy and radiotherapy [2]. The difficulty in precision treatment of gliomas results largely from its invasive and expansive growth, drug resistance, and recurrence. Since gliomas are highly dependent on vascularization for their growth, inhibition of the abundant tumor angiogenesis and blood supply are potential targets for the treatment of gliomas [3, 4]. However, previous studies have indicated that treatment with antiangiogenic drugs alone is insufficient to improve the prognosis of glioma patients [5, 6, 7], implying that other escape mechanisms that preserve tumor angiogenic processes might occur to ensure the blood supply to glioma tumors.

Vasculogenic mimicry (VM), a tumor vascular paradigm independent of angiogenesis, is an alternative process of vasculature formation by tumor cells that provides an additional blood supply for tumor growth and metastasis [8, 9]. Recent studies indicates that VM formation is closely associated with tumorigenesis, tumor development, and metastasis in various tumors, including colorectal cancer, hepatocellular carcinoma (HCC), non‐small‐cell lung cancer (NSCLC), breast cancer, and glioma [10, 11, 12, 13, 14]. In this context, VM formation probably results in failure of antiangiogenic therapy, as drug resistance or angiogenic rebound rapidly occurs once treatment is terminated [7, 15, 16]. To date, several signaling molecules, including ERK/PI3K/MMPs, VEGFA/VEGFR2, KAI1/E‐cadherin, and vimentin, have been identified to be involved in VM formation [17, 18, 19, 20, 21]. Thus, it is important to understand the mechanisms of VM formation to improve the effect of antiangiogenic treatment for tumor patients.

Neural cell adhesion molecule L1 (L1CAM, also termed L1), which is usually regarded as an important regulator in neural development and functions of adult brain [22, 23, 24, 25, 26, 27], was recently determined to be strongly associated with an aggressive tumor phenotype, high tumor grade, chemoresistance, metastasis, and poor patient prognosis in various tumors [28, 29, 30, 31, 32, 33, 34]. It is known that the adhesion capability between cancer cells and the extracellular matrix is associated with metastasis in malignant tumors. As a neural cell adhesion molecule, L1 was found to not only remodel the extracellular matrix but also enhance tumor metastasis in many malignancies [35]. Notably, a previous study demonstrated that L1 expression was highly expressed in blood vessels of human pancreatic carcinomas and in vessels of other tumors, whereas the inhibition of L1 expression in tumor‐bearing mice could suppress angiogenesis, improve vascular stabilization, and reduce tumor metastasis [36], suggesting that L1 might affect the tumor vasculature.

Here we identified L1 as a potential regulator of VM formation in glioma. The results indicated that L1 overexpression could enhance VM formation by promoting the invasive abilities of glioma cells. Consistent with this finding, miRNA‐seq analysis identified that the miR‐143‐3p‐regulated Hexokinase 2 (HK2) was involved in L1‐mediated VM formation by glioma cells. In addition, the blockade of L1/HK2 cascade significantly impairs the resistance of antiangiogenic treatment *in vivo* and *in vitro*. Further investigations demonstrated that the activation of phosphoinositide 3‐kinase (PI3K)/AKT signaling pathways was also associated with the process of L1‐mediated VM formation by glioma cells. Thus, our findings uncover a new role of L1 in regulating VM formation in glioma, which might provide therapeutic strategies for improving the prognosis of glioma patients.

## 2 Materials and methods

### 2.1 Cell culture and treatments

The human glioma cell lines U87 and T98 were purchased from the China Center for Type Culture Collection (Shanghai, China). All cells were maintained in Dulbecco's modified Eagle's medium (11965‐092, DMEM, Gibco, Thermo Fisher Scientific, Waltham, MA, USA) supplemented with 10% fetal bovine serum (10100147C, FBS, Gibco) and a streptomycin/penicillin antibiotic mixture (15140‐122, Gibco) at 37 °C in 5% CO_2_. The primary glioma cell culture protocol was slightly modified from a previously reported protocol [37, 38]. The glioma cell lines U87 and T98 were authenticated by short tandem repeat (STR) profiling of 15 loci and the amelogenin sex determination (X or XY) method (DG4640, Promega, Madison, WI, USA) according to previously described methods [39]. The authenticated primary glioma cell line (GBM1), obtained from Procell (Wuhan, China), was derived from GBM surgical specimens and maintained in primary serum‐free cultures grown on laminin [40]. Before the following experiment, the GBM1 cells were cultured in DMEM medium, supplemented with 10% FBS, streptomycin/penicillin antibiotic mixture, and 2 mm l‐glutamine (25030081, Gibco) at 37 °C with 5% CO_2_. For inhibiting AKT signaling pathway, the cells were treated with 10 μm MK‐2206 (HY‐108232, MedChemExpress, Monmouth Junction, NJ, USA), a specific AKT inhibitor, for 24 h before the following experiments.

### 2.2 Overexpression and knockdown

Overexpression and knockdown of L1 were induced by a lentiviral system. Briefly, hL1CAM, shL1CAM, and the control vector were designed and purchased from VectorBuilder (Suzhou, China). The corresponding plasmids were transfected into HEK293T cells by using Transintro EL Transfection Reagent (FT201‐01, TransGen Biotech, Beijing, China) to generate lentiviral particles. Thereafter, the supernatant containing viruses was harvested by centrifugation at 4000 × ***g*** for 10 min. The stable overexpression (L1‐OE) or knockdown (shL1) of L1 and vector control (VC) groups in U87, T98, and GBM1 glioma cell lines were established by infecting the virus‐containing pellets at 37 °C for 48 h and subsequently selected with 3 μg·mL^−1^ puromycin for 7 days.

To overexpress or knockdown miR‐143‐3p, the miR‐143‐3p mimic (miR‐143‐3p) and its negative control (NC), miR‐143‐3p inhibitor (in‐miR‐143‐3p) and its negative control (in‐NC), which were designed and purchased from RiboBio (Guangzhou, China), were transfected into different glioma cell lines by using Transintro EL Transfection Reagent (TransGen Biotech). In addition, to inhibit HK2 expression in glioma cells small interfering RNAs (siRNAs) targeting HK2 (siHK2‐1 and siHK2‐2) and its scrambled siRNA control (siNC) were transfected with Transintro EL Transfection Reagent (TransGen Biotech). All primer sequences are listed in Table S1.

### 2.3 Tissue microarray (TMA) analysis

The paraffin‐embedded commercialized tissue microarrays (*n* = 180, HBraG180Su01, Outdo Biotech Ltd, Shanghai, China; *n* = 85, N095Ct01, Bioaitech, Xi'an, Shaanxi Province, China) containing glioma specimens was subjected to immunohistochemistry staining applying corresponding primary antibodies. The TMA stained with human anti‐L1CAM antibody (BioLegend, San Diego, CA, USA) and the quantification of immunoreactive staining were evaluated using image‐pro plus software (Media Cybernetics, Rockville, MD, USA), according to a previous report [41]. Briefly, the optical density of the images of all tissue scores, which were photographed with a constant set of imaging parameters, were analyzed using image‐pro software. After adjusting the background and color intensity range in a representative high‐immunoreactivity image, the black and incident levels (the white background and the optical density of the maximal positive staining) from the immunostained and blank regions of the images were determined, respectively. The selection of intensity range was based on a histogram, with a maximum of saturation (S) and intensity (I) and a range within most of the brown AEC staining of hue (H), expect for blue nuclear counterstaining. The setting parameters were applied to the analyses of immunohistochemistry (IHC) staining. The integrated optical density (IOD) represents the immunoreactivity of the candidate protein in tumor tissues. The mean optical density was determined in the selected area (IOD/unit area) after defining the area of interest (AOI). The median value of the immunoreactive score was chosen as the cutoff criterion to define high‐ and low‐expression subgroups. All patients provided written informed consent before enrollment in this study (SHYJS‐CP‐1801003). The ethics approval was approved by the Medical Ethics Committee of Shantou University Medical College (SUMC‐2018‐25). The study methodologies conformed to the standards set by the Declaration of Helsinki.

### 2.4 Xenografts in BALB/c‐nu mice and drug treatment

The BALB/c‐nu mice were obtained from Nanjing Biomedical Research Institute (Nanjing, China), and housed under standard pathogen‐free conditions at 25 °C under a 12‐h light–dark cycle with food and water, according to the guidelines of the Animal Care Committee of Shantou University Medical College. A total of 5 × 10^5^ dissociated cells from L1 overexpression and control groups were injected into the right frontal lobe in 3‐week‐old male BALB/c‐nu mice (*n* = 3 for each group). Three weeks later, all xenografted mice were sacrificed after isoflurane treatment, and the tumor‐bearing tissues derived from the xenografted mice were fixed by using transcranial perfusion with 4% paraformaldehyde (PFA) and paraffin‐embedded for the following IHC.

For drug treatment, U87 cells were injected subcutaneously (3 × 10^6^) into the flanks of BALB/c‐nu mice; when the tumor volume reached about 100‐mm^3^, mice were randomly divided into four groups (*n* = 4 for each group), and treated (three times a week, i.p.) with drugs: bevacizumab (100 μg per mouse, A2006, Selleck, Houston, TX, USA), anti‐L1 neutralizing monoclonal antibody (anti‐L1, 300 μg per mouse, UJ127.11, InVivo BioTech Services, Hennigsdorf, Germany) [39], a combination of bevacizumab and anti‐L1 or the vehicle control (phosphate‐buffered saline [PBS]). Tumor growth was monitored using a Vernier caliper at days 9, 12, 15, 18, and 21. At day 21, the tumors derived from different groups were excised and weighed. The tumor sizes were calculated using the following formula: [length (mm) × width (mm) × width (mm) × 0.5]. In addition, U87 cells transduced with luciferase were also intracranially injected (5 × 10^5^) into the right frontal lobe. The randomly selected xenograft mice were divided into four drug‐treated groups (*n* = 3 for each group, three times a week, i.p.), including bevacizumab, anti‐L1, the combination of bevacizumab and anti‐L1, and control groups. The different groups were monitored by detecting bioluminescent activity using an IVIS *in vivo* imaging system (PerkinElmer, Waltham, MA, USA) at days 7 and 21. All animal experiments in this study were approved by the Shantou University Medical College Animal Committee (SUMC2019‐001 and SUMC2022‐225).

### 2.5 Immunohistochemistry (IHC)

The TMA or paraffin‐embedded tissues were baked at 55–60 °C for 2 h and then dewaxed twice in xylene for 10 min. After blocking the slides with 5% bovine serum albumin (BSA) for 1 h at 37 °C, the samples were incubated with the primary antibodies at 4 °C overnight. Thereafter, the slices were washed three times with PBS for 5 min, and the secondary antibody was added and incubated at room temperature for 3 h. The stained slides were then detected by using the avidin‐biotin complex method (Dako, Glostrup, Denmark), visualized with the AEC method (Zhongshan Goldbridge Biotechnology, Beijing, China), and evaluated with image‐pro plus software (Media Cybernetics). The sources of the antibodies are listed in Table S2.

### 2.6 Immunofluorescence (IF) staining

A total of 1 × 10^5^ cells were seeded on coverslips in a 24‐well plate overnight. The cells were fixed with 4% PFA for 15 min and washed with PBS three times for 5 min each time. After treatment with 0.1% Triton X‐100 for 2 min, the samples were blocked with 3% BSA at room temperature for 1 h and separately incubated with the primary antibodies. After three washes with PBS at room temperature for 15 min, the secondary antibody and DAPI were added and incubated at room temperature for 1 h. The samples were then observed and imaged under a confocal laser scanning microscope (LSM800, Zeiss, Oberkochen, Germany). The sources of the antibodies are listed in Table S2.

### 2.7 Total RNA extraction and quantitative real‐time polymerase chain reaction (qRT‐PCR analysis)

Total RNA was extracted from different cell lines with TRIzol Reagent (15596026, Invitrogen, Carlsbad, CA, USA) according to the manufacturer's instructions. The extracted total RNA was reverse transcribed with a Mir‐X miRNA FirstStrand Synthesis Kit (638313, TaKaRa Bio, Kusatsu, Japan) and amplified by using SYBR Green PCR Master Mix (4309155, GenStar, Beijing, China). The relative levels of miRNAs were evaluated with cfx‐managertm v3.2 software (Bio–Rad Laboratories, Hercules, CA, USA) and normalized to U6 using the 2^−ΔΔCt^ method. All primer sequences are listed in Table S1.i

### 2.8 Western blot analysis

Protein samples were lysed by using RIPA solution (R0010, Solarbio, Beijing, China) containing protease inhibitor tablets (539133, Solarbio) at 4 °C for 15 min. The lysates were separated on 12% sodium dodecyl sulfate‐polyacrylamide gels (SDS/PAGE) and transferred to polyvinylidene fluoride (PVDF) membranes (Millipore, Billerica, MA, USA). The blots were blocked in 5% BSA and incubated with the appropriate primary antibodies at 4 °C overnight. After washing, the blots were incubated with the corresponding secondary antibodies at room temperature for 2 h. The signals were visualized using an ECL western blot kit (32106, Pierce, Thermo Fisher Scientific). The pixel intensities were measured with imagej software (National Institutes of Health, Bethesda, MD, USA). The sources of the antibodies are listed in Table S2.

### 2.9 Wound healing and transwell assays

Cells were cultured in DMEM with 10% FBS in 6‐well plates until confluence reached about 80%, and then maintained in serum‐free DMEM medium after scratching with a sterile 10 μL pipette tip. The width of wounds was measured at different timepoints (0 and 24 h). For the determination of the transwell assay, a total of 5 × 10^4^ cells were seeded into the chamber coated with Matrigel (1 : 20, 356231, BD Biosciences, San Jose, CA, USA). The upper chambers of each plate were added with serum‐free DMEM medium, and the bottom chambers were filled with DMEM supplemented with 10% FBS. After 16 h of incubation, the cells on the upper surface were removed with a cotton swab, and the membranes were fixed with 4% PFA and stained with 0.1% crystal violet. The invasive cells were counted under a phase‐contrast microscope.

### 2.10 Tube formation angiogenesis assay

The ice‐cold Matrigel was added to a 96‐well plate and incubated at 37 °C for 30 min for solidification. Cells suspended in medium with 10% FBS were seeded in the solidified Matrigel and incubated for 3–6 h. For drug treatment, bevacizumab (1 μg·mL^−1^) and/or anti‐L1 neutralizing monoclonal antibody (1 μg·mL^−1^) were respectively treated in L1‐OE, shL1, and VC groups in different glioma cell lines for 48 h. The images were acquired by using a Zeiss Axiovert 200M microscope. Tube formation was quantified by measuring the total number of tubes using imagej software.

### 2.11 Global transcriptome sequencing (RNA‐seq) and miRNA‐seq analysis (miRNA‐seq)

Total RNA from L1‐knockdown (shL1) and vector control (VC) groups of U87 cells was extracted using Trizol Reagent and sequenced by the BGI company (Shenzhen, China). The mRNA‐seq library was prepared for sequencing using standard Illumina protocols. In brief, the total RNA samples were denatured and isolated from the sample of interest. The mRNA was enriched using Oligo (dT) beads, and the cleaved mRNA fragments were converted to cDNA using reverse transcriptase and random priming. The second strand of cDNA was synthesized using DNA polymerase I and RNase H. Thereafter, each sample was ligated to a sequencing adapter to obtain the mRNA‐seq library. The fragment and concentration of the library were detected using Agilent 2100 Bioanalyzer (Agilent Technologies, Santa Clara, CA, USA). RNA‐seq was double‐end sequenced with a read length of 150 bp and the depth of sequencing was 6G. Genes with a |log2 foldchange| ≥ 2 and *P* < 0.05 were considered differentially expressed. The gene ontology analysis was carried out by the online program metascape (http://metascape.org).

miRNA‐seq analysis was performed by BGI Technology with total RNA from the L1‐OE and vector control groups of U87 cells. The small RNA regions corresponding to the 18–30 nt were isolated by agarose gel electrophoresis. The recovered small RNAs were then ligated to a modified adapter and subsequently transcribed into cDNA with SuperScript II Reverse Transcriptase (Invitrogen). Thereafter, the cDNA fragments were enriched by PCR amplification and measured using the Agilent 2100 bioanalyzer. The ligation PCR products were sequenced using the BGISEQ‐500 platform (BGI). miRNA‐seq was single‐end sequenced with a read length of 50 bp and the depth of sequencing was 20 m. The significantly differentially regulated miRNAs were further confirmed by qRT‐PCR. The target genes of the miRNAs were predicted by using the TargetScan, miRDB, miRTarBase, and TarBase databases. All primer sequences are listed in Table S1.

### 2.12 Neurosphere formation assay

The cells stably transfected with L1‐OE and vector control plasmids were seeded in 24‐well plates at 2 × 10^4^ for 12–18 h. The L1 overexpressed glioma cells were pretreated with miR‐143‐3p mimic or siHK2‐2 for 24 h. Thereafter, all cells were cultured in serum‐free neural stem cell medium containing DMEM/F12 medium (11320‐033, Gibco), supplemented with 20 ng·mL^−1^ bFGF (HY‐P7179, MedChemExpress), 20 ng·mL^−1^ EGF (HY‐P7109, MedChemExpress), 1% B27 (17504‐044, Gibco), and the streptomycin/penicillin antibiotic mixture. The cells were cultured until several primary tumor spheres were visible under microscopy. The primary tumor spheres were dissociated into single cells, and seeded in 24‐well plates in 1 mL per well of serum‐free neural stem cell medium. The culture medium was changed every 3 days. The neurospheres from secondary spheres derived from single cells of primary spheres were determined using an Axio Observer A1 microscope (Zeiss).

### 2.13 Luciferase assay

To determine whether miR‐143‐3p directly targets the HK2 3'UTR, the binding sites of the wildtype (wt) or mutated 3'UTR of HK2 were amplified and inserted into the PGL6‐miR vector (D2106‐100 μg, Beyotime, Beijing, China). The constructs of the wildtype control (HK2‐wt) or luciferase plasmids containing the mutated *HK2* 3'UTR binding site (HK2‐mut) were transfected into U87 cells. After 48 h, luciferase activities were measured with a Dual‐Luciferase Reporter Assay System (RG027, Beyotime).

### 2.14 Quantification and statistical analysis

All statistical analyses were performed using spss 22.0 software (SPSS, Chicago, IL, USA) or graphpad prism 8.0 software (GraphPad Software, San Diego, CA, USA). All experiments were conducted with at least three replicates. The correlation between the expression of L1CAM and the regulators of glioma invasive or VM formation (e.g. MMP2, MMP9, CD31, and CD34) was evaluated using Spearman correlation analyses. To calculate statistical significance, two‐tailed Student's *t*‐test or one‐way analysis of variance (ANOVA) followed by Tukey's multiple comparisons test was used to determine the significance of differences between the indicated groups. The differences in patients' survival curves between different subgroups were evaluated by Kaplan–Meier analyses and the statistical significance of differences between the survival curves was assessed with a log‐rank test. Data were expressed as mean ± SEM. *P < 0.05, **P < 0.01, ***P < 0.001, and ****P < 0.0001 were regarded as statistically significant.

## 3 Results

### 3.1 L1 is highly expressed and positively associated with poor prognosis in patients with glioma

**Fig. 1:**
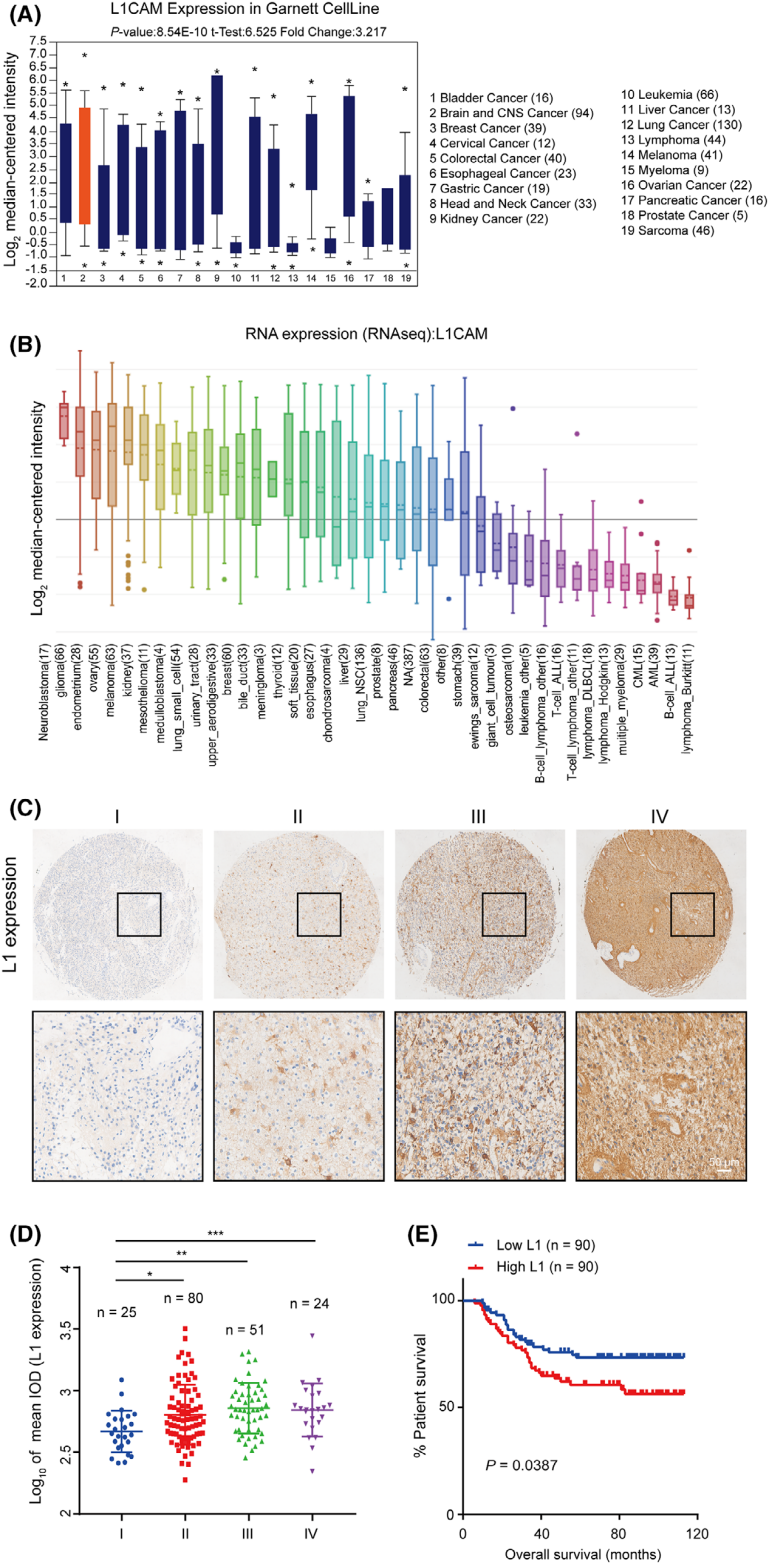
L1 expression is positively correlated with malignancy degree and prognosis in glioma patients. (A,B) The mRNA levels of L1 in various types of cancers from the Oncomine (A) and CCLE (B) databases. (C) Representative images of L1 expression in a tissue microarray of glioma specimens with I (*n* = 25), II (*n* = 80), III (*n* = 51), and IV (*n* = 24) grades. Scale bar, 50 μm. (D) The positive correlation between L1 expression and the malignancy of gliomas (*n* = 180). (E) Overall survival rates in higher or lower L1‐expressing specimens from glioma patients (*n* = 180). The survival curves were evaluated by Kaplan–Meier analyses and the statistical significance of differences between the survival curves was assessed with a log‐rank test. One‐way ANOVA followed by Tukey's multiple comparisons test was used to generate *P* values. Data expressed as mean ± SEM. *P < 0.05, **P < 0.01, and ***P < 0.001. L1, neural cell adhesion molecule L1.

To elucidate the clinical significance of L1 in patients with glioma, we first performed data mining to analyze the expression level of L1 in various tumors. We analyzed the mRNA expression of L1 in different cancers through the Oncomine database. The results showed that the mRNA levels of L1 were highly increased in brain and central nervous system (CNS) cancer (Fig. 1A). Further CCLE database analysis displayed the highest expression of L1 in neuroblastoma and glioma (Fig. 1B), suggesting that L1 might play a role in glioma tumorigenesis and tumor development.

To further show the potential roles of L1 in glioma, IHC analysis was performed to examine the correlation between L1 expression and malignancy/prognosis in human glioma microarray specimens (*n* = 180, HBraG180Su01, Outdo Biotech). The results indicated that the average L1 staining intensity was weak in low‐grade gliomas (*n* = 105; LGG; I/II grades), whereas high expression of L1 was detected in high‐grade gliomas (*n* = 75; GBM; III/IV grades; Fig. 1C). Quantification also demonstrated that L1 expression was positively correlated with malignancy (Fig. 1D) and prognosis (Fig. 1E) in glioma patients, which is also consistent with the concept that L1 expression is correlated with prognosis in endometrial cancer [42, 43]. In addition, as shown in Table S3, strong associations were observed between L1 expression and tumor grades (*P* = 0.001) and tumor recurrence (*P* = 0.003). Therefore, these results indicated that the functions of L1 were probably involved in tumorigenesis and tumor development in glioma patients.

### 3.2 L1 promotes glioma invasion by upregulating MMP2 and MMP9 expression

**Fig. 2:**
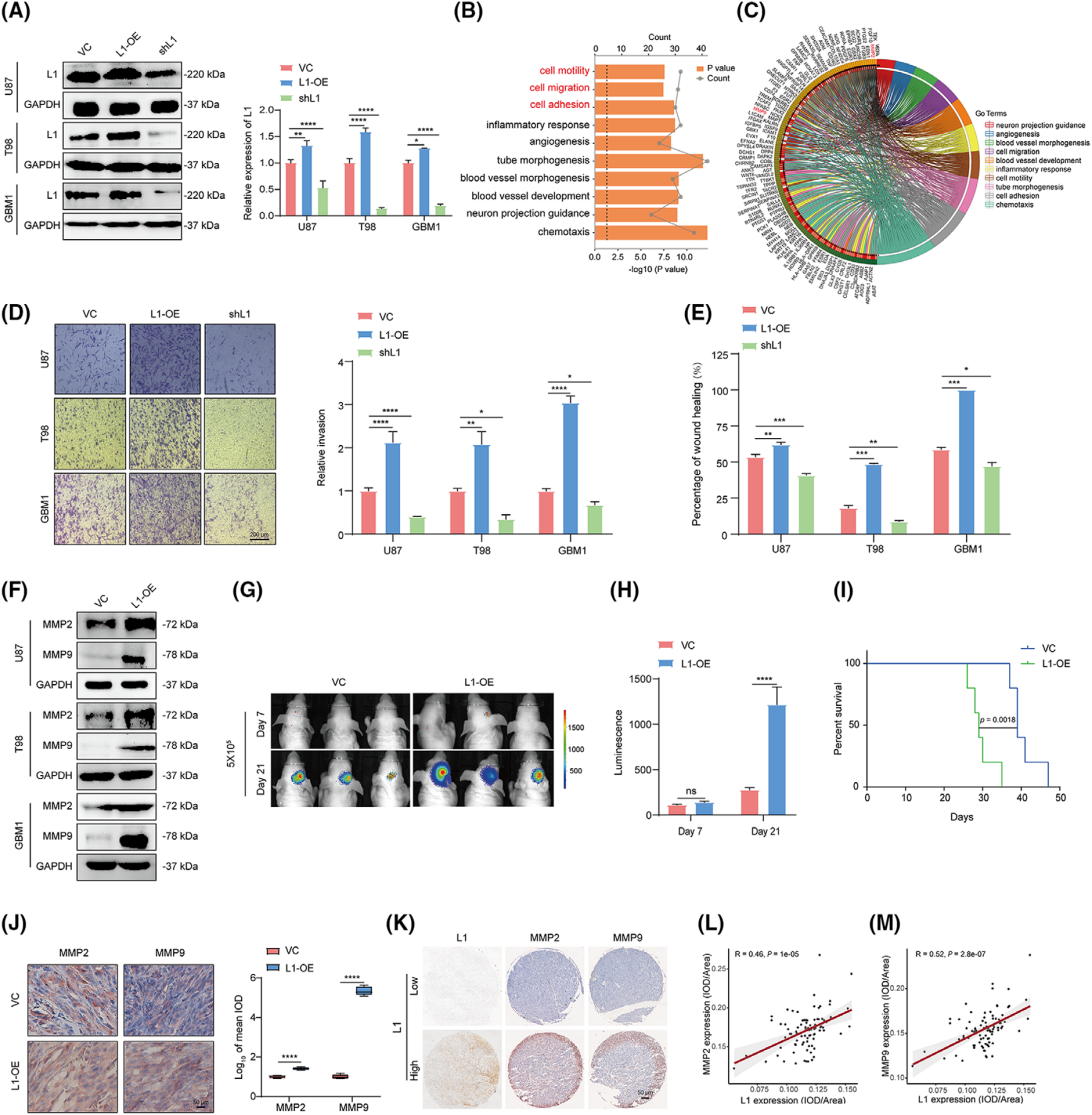
L1 overexpression enhances glioma invasion by upregulating MMP2 and MMP9 expression in glioma cells. (A) Overexpression and knockdown of L1 in U87, T98, and GBM1 glioma cell lines (*n* = 3 replicates). (B) GO analysis of the top 10 GO terms with DEGs between the L1‐knockdown and vector control groups of U87 cells. Data is visualized through the ggplot2 package and analyzed with the clusterprofiler package. (C) Chord diagram of DEGs in these top 10 GO terms. (D,E) The transwell (D) and wound‐healing (E) assay results show the effects of L1 expression on tumor invasion in the L1‐OE, shL1, and control groups of U87, T98, and GBM1 glioma cell lines (*n* = 3 replicates). Scale bar, 200 μm. (F) L1 overexpression induces upregulation of MMP2 and MMP9 expression in different glioma cell lines (*n* = 3 replicates). (G,H) Representative bioluminescent images (G) and the quantification (H) of intracranial xenografts derived from L1‐overexpressing (L1‐OE) and vector control (VC) U87 glioma cells (*n* = 3 for each group). (I) Kaplan–Meier survival curves of the xenograft mice bearing the L1‐OE glioma tumors (*n* = 3 for each group) with a log‐rank test. (J) Representative images from the different groups and quantification of tumor‐bearing tissues stained with anti‐MMP2 or anti‐MMP9 antibody in L1‐OE and vector control U87 xenograft‐bearing BALB/c‐nu mice (*n* = 3 for each group). Scale bar, 50 μm. (K) Representative images of IHC staining of L1, MMP2, and MMP9 in human glioma specimens (*n* = 85). (L,M) Spearman rank correlation analysis of L1 and MMP2 (L) or MMP9 (M) in human glioma specimens (*n* = 85). A two‐tailed Student's *t*‐test was used to generate *P* values. Data expressed as mean ± SEM. *P < 0.05, **P < 0.01, ***P < 0.001, and ****P < 0.0001. DEGs, differently expressed genes; GO, gene ontology; L1, neural cell adhesion molecule L1; L1‐OE, L1‐overexpressing; shL1, L1 knockdown; VC, vector control.

We first established stable L1‐overexpressing (L1‐OE) and L1‐silenced (shL1) U87, T98, and primary (GBM1) glioma cell lines (Fig. 2A). RNA‐seq analysis was then performed to identify the potential effects of L1 in U87 human glioma cells (shL1 vs. VC control). Gene ontology (GO) term enrichment revealed that glioma invasion is significantly regulated (marked in red, Fig. 2B). Furthermore, the differentially expressed genes (DEGs) analysis indicated that matrix metalloproteinases (MMPs) might be involved in this process (marked in red, Fig. 2C, Table S4), which is consistent with the previous reports that MMPs, including MMP2 and MMP9, are critical factors for metastasis and invasion in various tumors [44, 45]. As we expected, transwell and wound‐healing assays demonstrated that glioma invasion could be enhanced by overexpression of L1 but inhibited after knockout of L1 expression in glioma cells (Fig. 2D,E). In addition, L1 overexpression significantly induces the upregulation of MMP2 and MMP9 expression in U87, T98, and GBM1 cells (Fig. 2F), suggesting that L1 might promote tumor invasion through upregulating MMP2 and MMP9 in glioma cells.

To further investigate the effects of L1 on glioma invasion, orthotopically tumorigenicity showed that the sizes of tumors from BALB/c‐nu mice xenografted L1‐overexpressing U87 cells were much higher than those of the tumors from mice receiving untreated U87 cells (Fig. 2G,H). There were also significant differences in survival times of the mice between L1‐OE and the control groups (*P* = 0.0018, Fig. 2I). In addition, IHC analysis confirmed the upregulation of MMP2 and MMP9 expression in the tumors derived from the L1‐overexpressing U87 xenograft‐bearing mice (Fig. 2J). Importantly, in human glioma microarray specimens (*n* = 85, N095Ct01, Bioaitech), we found a positive correlation between L1 and MMP2/9 staining intensities in the glioma specimens (Fig. 2K–M), thus supporting the notion that MMP2 and MMP9 are involved in L1‐mediated glioma invasion.

### 3.3 L1 overexpression enhances VM formation by glioma cells

**Fig. 3:**
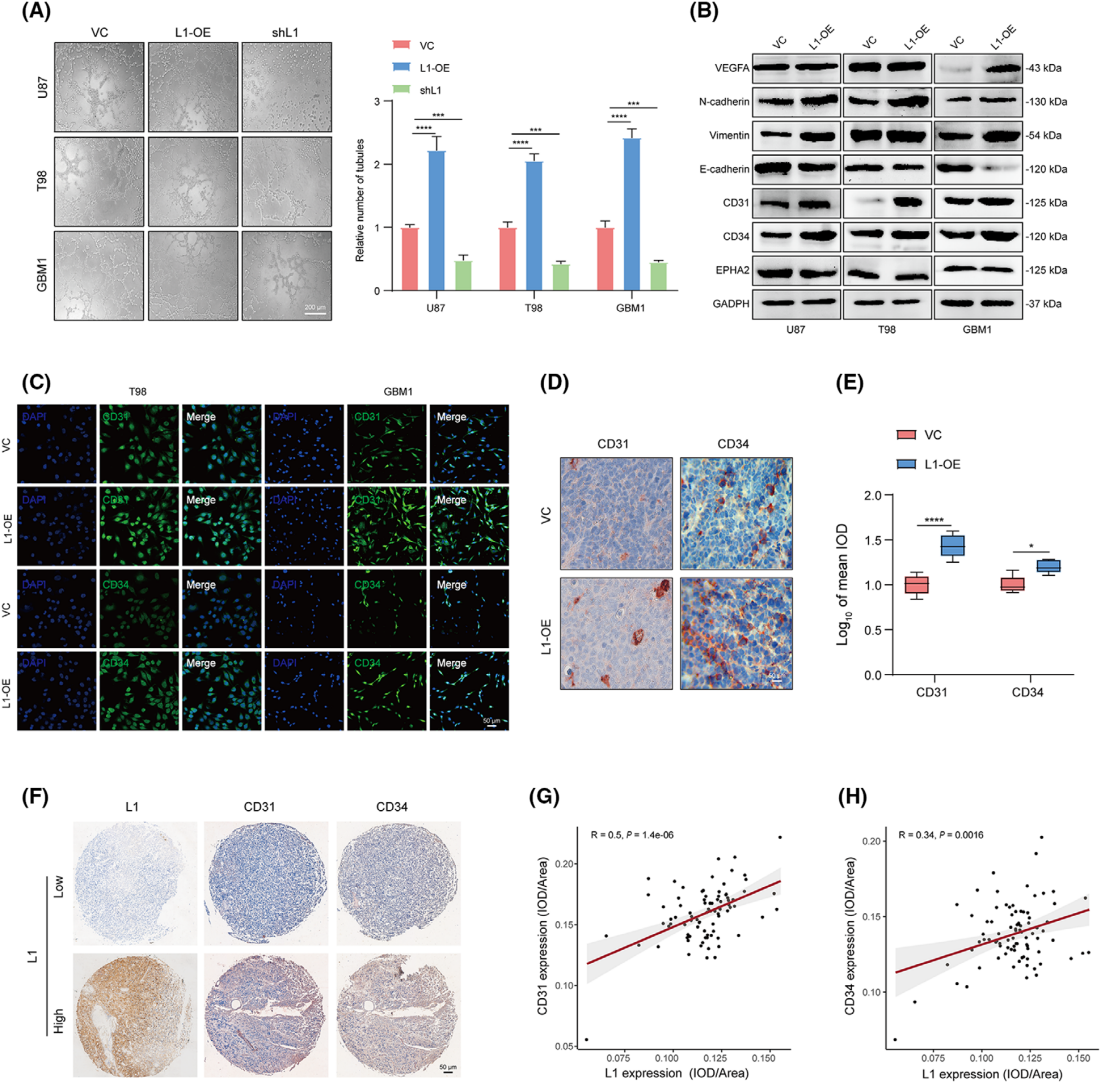
L1 overexpression promotes VM formation by glioma cells. (A) Representative images and quantification of tube formation in the L1‐OE, shL1, and control groups of U87, T98, and GBM1 glioma cell lines (*n* = 3 replicates). Scale bar, 200 μm. (B) Relative expression of key regulators of VM formation, including VEGFA, N‐cadherin, vimentin, E‐cadherin, CD31, CD34, and EPHA2, in the L1‐OE and control groups of different glioma cell lines (*n* = 3 replicates). (C) Representative images of the increasing fluorescent signals of CD31 and CD34 detected by anti‐CD31 or anti‐CD34 antibody (FITC, green) in L1‐OE and control groups of T98 and GBM1 cells (*n* ≥ 3 replicates). Scale bar, 50 μm. (D,E) Representative images (D) and quantification (E) of tumor‐bearing tissues stained with anti‐CD31 or anti‐CD34 antibody in L1‐OE and vector control U87 xenograft‐bearing BALB/c‐nu mice (*n* = 3 for each group). Scale bar, 50 μm. (F) Representative images of IHC staining of L1, CD31, and CD34 in human glioma specimens (*n* = 85). Scale bar, 50 μm. (G,H) Spearman rank correlation analysis of L1 and CD31 (G) or CD34 (H) in human glioma specimens (*n* = 85). A two‐tailed Student's *t*‐test was used to generate *P* values. Data expressed as mean ± SEM. *P < 0.05, ***P < 0.001, and ****P < 0.0001. EPHA2, EPH receptor A2; L1, neural cell adhesion molecule L1; L1‐OE, L1‐overexpressing; shL1, L1 knockdown; VC, vector control; VEGFA, vascular endothelial growth factor A.

Since multiple molecular mechanisms, including MMPs, vascular endothelial growth factor receptor (VEGFR1), and hypoxia‐inducible factor (HIF)‐1α, have been reported to participate in VM formation, which is linked to tumor invasion and metastasis [46, 47], we attempted to confirm whether the aberrant expression of L1 is associated with the regulation of VM formation in glioma. Tube formation demonstrated that L1 overexpression enhanced VM formation in U87, T98, and GBM1 cells. In contrast, the suppression of L1 expression significantly inhibited tube formation in these three groups of glioma cells (Fig. 3A). Additionally, we found that L1 overexpression resulted in upregulation of several VM formation‐related factors, including CD31, CD34, VEGFA, vimentin, and N‐cadherin, whereas it downregulated E‐cadherin expression in glioma cells (Fig. 3B). However, the expression of EPH receptor A2 (EphA2), a potential indicator of VM formation, was not remarkably regulated after overexpressing L1 in glioma cells, suggesting that the EphA2 signal might not be associated with L1‐mediated VM formation in glioma. Similarly, immunofluorescence staining further confirmed that the expression of CD31 and CD34, which are the key biomarkers in the VM formation process [48, 49], was significantly increased in L1‐overexpressing T98 and GBM1 cells (Fig. 3C). Furthermore, IHC analysis confirmed the upregulation of CD31 and CD34 expression in tumor tissues derived from L1‐overexpressing U87 xenograft‐bearing BALB/c‐nu mice (Fig. 3D,E). Notably, we determined the expression of CD31 and CD34 in human glioma specimens (*n* = 85, N095Ct01, Bioaitech). The results indicated that L1 expression is positively correlated with CD31/CD34 expression in glioma (Fig. 3F–H), suggesting that L1 overexpression could affect VM formation in glioma.

### 3.4 Identification of the potential downstream targets in L1‐mediated VM formation

**Fig. 4:**
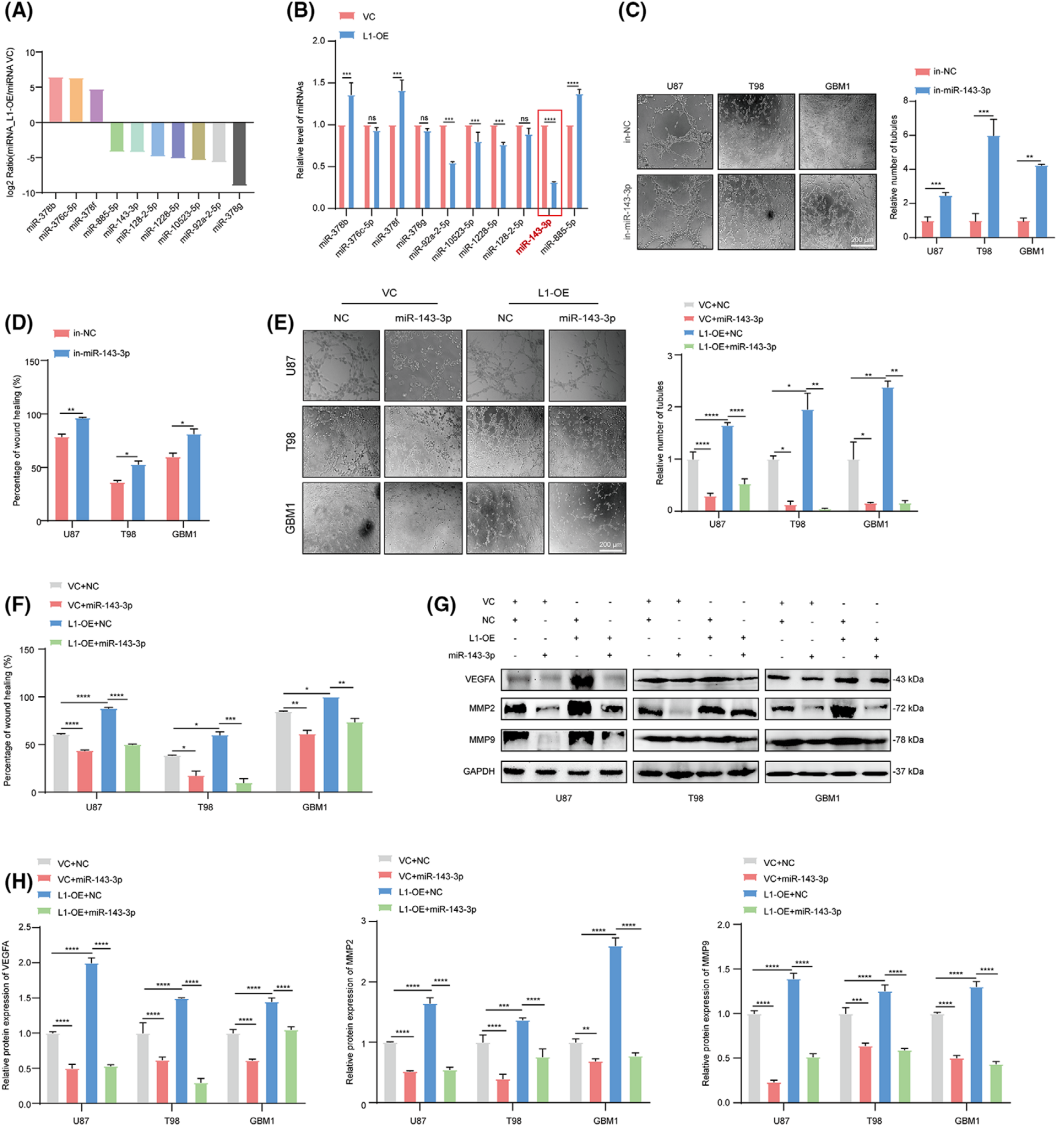
miRNA‐143‐3p is involved in L1‐mediated VM formation in glioma. (A) The top 10 differentially expressed miRNAs in L1‐overexpressing and vector control U87 glioma cells. (B) The qPCR confirmation of miRNA‐seq identified the top 10 regulated miRNAs in L1‐OE and VC groups in U87 glioma cells (*n* = 3 replicates). (C) Representative images and quantification of tube formation in the in‐miR‐143‐3p and in‐NC groups of U87, T98, and GBM1 cell lines (*n* = 3 replicates). (D) Treatment with in‐miR‐143‐3p suppresses invasion in different glioma cell lines (*n* = 3 replicates). (E,F) Representative images and quantification of tube formation and tumor invasion in the L1‐OE and vector groups of different glioma cell lines with or without miR‐143‐3p mimic transfection (*n* = 3 replicates). (G,H) Determination (G) and quantification (H) of MMP2, MMP9, and VEGFA expression in miR‐143‐3p mimic‐treated L1‐OE and vector control groups of U87, T98, and GBM1 glioma cell lines (*n* = 3 replicates). In (B–D), a two‐tailed Student's *t*‐test was used to generate *P* values. In (E–H), one‐way ANOVA followed by Tukey's multiple comparisons test was used to generate *P* values. Data expressed as mean ± SEM. *P < 0.05, **P < 0.01, ***P < 0.001, and ****P < 0.0001. in‐miR‐143‐3p, miR‐143‐3p inhibitor; in‐NC, miR‐143‐3p inhibitor negative control; L1, neural cell adhesion molecule L1; L1‐OE, L1‐overexpressing; miR‐143‐3p, miR‐143‐3p mimic; MMP2, matrix metalloproteinase 2; MMP9, matrix metalloproteinase 9; NC, miR‐143‐3p mimic negative control; ns, not significant; VC, vector control; VEGFA, vascular endothelial growth factor A.

It is known that miRNAs are closely linked to the regulation of tumor angiogenesis [50], tumorigenesis [51], and tumor invasion and metastasis [52]. In this context, the top 10 up‐ and downregulated miRNAs, which might be involved in VM formation and tumor invasion in glioma, were identified using miRNA‐seq in L1‐overexpressing and vector control U87 cells (Fig. 4A). Thereafter, further qPCR assay revealed that miR‐143‐3p was the most significantly regulated in L1‐overexpressing U87, T98, and GBM1 cell lines (red mark, >2‐fold change, *P* < 0.05, Figs 4B and S1A,B). In addition, the tube formation and wound‐healing assays showed that VM formation and tumor invasion were significantly promoted after inhibiting miR‐143‐3p expression in glioma cells (Fig. 4C,D), suggesting that miR‐143‐3p is involved in L1‐mediated VM formation by glioma cells.

To further confirm the effects of miR‐143‐3p on L1‐mediated VM formation, we next examined the VM formation capability in the L1‐overexpressing and control groups of glioma cells. The results indicated that the transfection of the miR‐143‐3p mimic could significantly suppress L1‐enhanced VM formation and tumor invasion in the U87, T98, and GBM1 glioma cell lines (Fig. 4E,F). In addition, western blot analyses showed that the expression of both angiogenic factors (VEGFA) and mesenchymal markers (MMP2 and MMP9), which are known as critical regulators of VM formation, was downregulated in miR‐143‐3p mimic‐transfected L1‐overexpressing and vector control groups in these three glioma cell lines (Fig. 4G,H), indicating that miR‐143‐3p could reverse L1‐mediated VM formation by regulating VEGFA, MMP2, and MMP9 expression in different glioma cell lines.

### 3.5 miR‐143‐3p directly targets the HK2‐3'UTR binding site in glioma cells

**Fig. 5:**
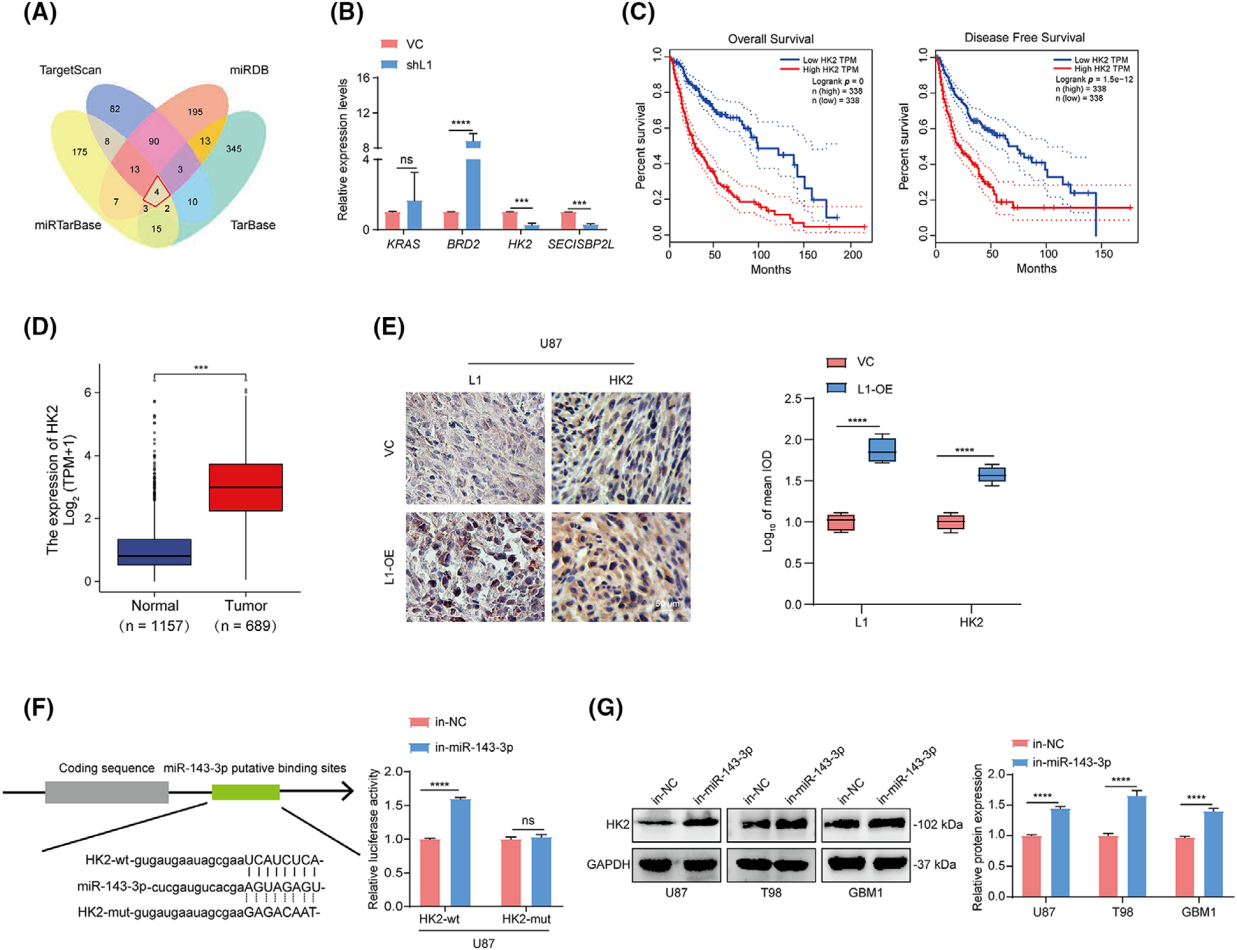
miR‐143‐3p directly targets the HK2‐3'UTR binding site in glioma cells. (A) The identification of the overlapping miR‐143‐3p‐targeted genes in the TargetScan, miRDB, miRTarBase, and TarBase databases. (B) qRT‐PCR confirmation of the overlapping genes in the shL1 and VC groups of U87 glioma cells (*n* = 3 replicates). (C) The overall survival and disease‐free survival rates of glioma patients with high and low HK2 expression from the GEPIA database (*n* = 338). The differences in patients' survival curves between different subgroups were evaluated by Kaplan–Meier analyses and the statistical significance of differences between the survival curves was assessed with a log‐rank test. (D) The evaluation of HK2 expression in glioma specimens (*n* = 689) and adjacent noncancerous tissues (*n* = 1157) in the UCSC Xena database. (E) Representative images and quantification of tumor‐bearing tissues stained with anti‐L1 or anti‐HK2 antibody in L1‐OE and vector control U87 xenograft‐bearing BALB/c‐nu mice (*n* = 3 for each group). Scale bar, 50 μm. (F) A luciferase reporter assay was performed in U87 cells cotransfected with HK2‐wt or HK2‐mut and in‐miR‐143‐3p and in‐NC (*n* = 3 replicates). (G) Determination and quantification of HK2 expression in in‐miR‐143‐3p‐ and in‐NC‐treated U87, T98, and GBM1 glioma cell lines by western blot analysis (*n* = 3 replicates). A two‐tailed Student's *t*‐test was used to generate *P* values. Data expressed as mean ± SEM. ***P < 0.001, ****P < 0.0001. HK2‐mut, the mutated 3'UTR sequence of *HK2* gene; HK2‐wt, the wildtype 3'UTR sequence of *HK2* gene; in‐miR‐143‐3p, miR‐143‐3p inhibitor; in‐NC, miR‐143‐3p inhibitor negative control; L1‐OE, L1‐overexpressing; ns, not significant; shL1, L1 knockdown; VC, vector control.

Considering that miR‐143‐3p is involved in L1‐enhanced VM formation by glioma cells, we hypothesized that an miRNA regulatory network at the posttranscriptional level might be responsible for this process. To identify the possible direct target genes of miR‐143‐3p, we utilized four bioinformatic databases: TargetScan, miRDB, miRTarBase, and TarBase, to predict the potential miR‐143‐3p‐targeted genes. Four genes, *KRAS, BRD2, HK2*, and *SECISBP2L*, were identified to potentially bind to miR‐143‐3p in the set of overlapping genes from these four datasets (red frame, Fig. 5A). Further qPCR analysis confirmed that the inhibition of L1 expression could induce downregulation of HK2 and SECISBP2L expression and upregulation of BRD2 expression, but did not affect KRAS expression in U87 cells (Fig. 5B). In addition, prognostic analyses indicated that only HK2 expression was positively correlated with poor prognosis (Figs 5C and S2A–D) and malignancy (Fig. 5D) in glioma patients in the GEPIA and UCSC Xena databases, respectively. Similarly, we also found high expression of HK2 in tumors derived from mice implanted with L1‐overexpressing U87 cells (*n* = 3 mice per group; Fig. 5E), suggesting that elevated L1 expression induces upregulation of HK2 expression *in vivo*.

To confirm whether miR‐143‐3p can directly bind to the 3'UTR of the *HK2* gene, a luciferase activity assay was performed to determine the miR‐143‐3p‐targeted HK2 3'UTR in glioma cells. The results demonstrated that the relative luciferase activity was increased after treatment with the miR‐143‐3p inhibitor (in‐miR‐143‐3p) in U87 cells transfected with the wildtype 3'UTR sequence of the *HK2* gene (HK2‐wt) but not the mutated 3'UTR sequence of the *HK2* gene (HK2‐mut; Fig. 5F). In addition, western blot analysis showed that HK2 expression was significantly upregulated in in‐miR‐143‐3p‐treated U87, T98, and GBM1 glioma cells (Fig. 5G), implying that miR‐143‐3p could regulate HK2 expression by targeting the 3'UTR of the *HK2* gene in glioma.

### 3.6 Blockade of the L1/HK2 cascade could negatively regulate L1‐mediated VM formation by glioma cells

To elucidate the potential roles of the L1/HK2 cascade in glioma invasion and VM formation, we activated or blocked the L1/HK2 cascade by inhibiting the expression of miR‐143‐3p or HK2 using an miR‐143‐3p inhibitor or HK2 siRNAs (siHK2‐1 and siHK2‐2) in U87, T98, and GBM1 glioma cell lines, respectively (Fig. S3A,B). As we speculated, the enhancement of glioma invasion and VM formation induced by the inhibition of miR‐143‐3p could be significantly reversed after downregulation of HK2 expression in U87, T98, and GBM1 glioma cell lines (Fig. S3C,D). Furthermore, after examining L1 expression in miR‐143‐3p‐pertubed glioma cells, we found that the activation of the L1/HK2 cascade failed to regulate the expression of L1 in glioma cells (Fig. S3E), suggesting that the regulation of miR‐143‐3p or HK2 can only affect tumor invasion and VM formation, but not be sufficient to regulate L1 expression through a positive feedback regulatory loop in glioma cells.

**Fig. 6:**
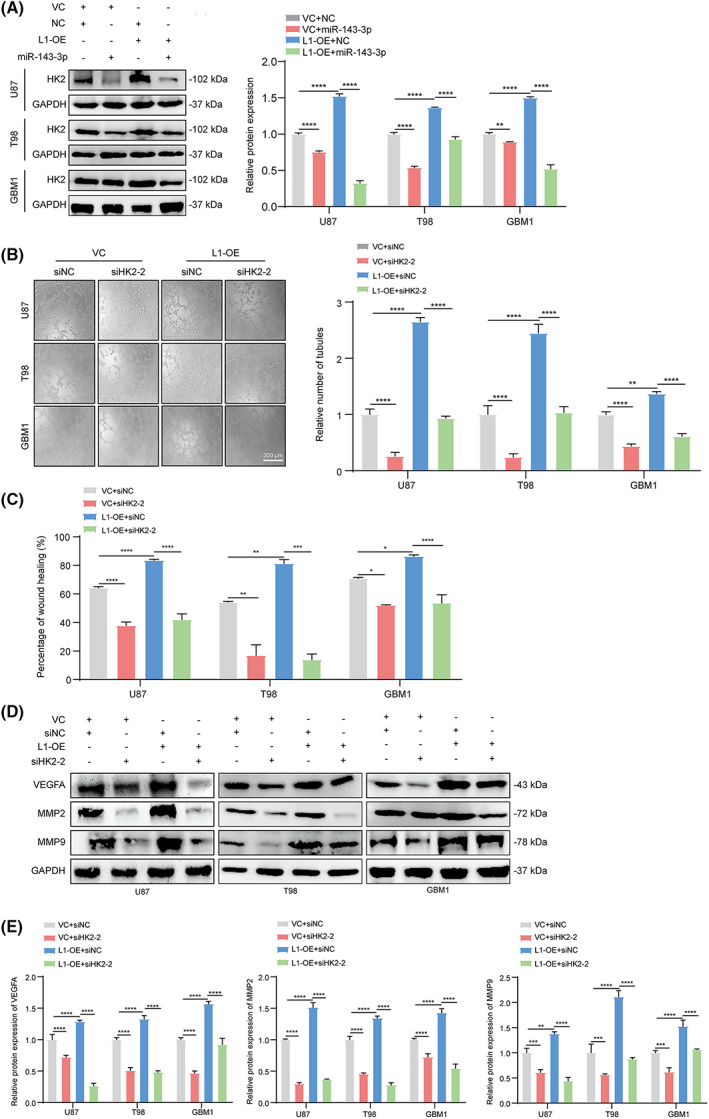
miR‐143‐3p‐regulated HK2 is involved in L1‐mediated VM formation by glioma cells. (A) Western blot examination and quantification of HK2 expression in miR‐143‐3p mimic‐treated L1‐OE and vector control groups of U87, T98, and GBM1 glioma cell lines (*n* = 3 replicates). (B) Representative images and quantification of tube formation in the L1‐OE and vector groups of different glioma cell lines with or without HK2 siRNA transfection (*n* = 3 replicates). Scale bar, 200 μm. (C) Knockdown of HK2 expression suppresses glioma invasion in the L1‐OE and vector control groups of different glioma cell lines (*n* = 3 replicates). (D,E) Determination (D) and quantification (E) of MMP2, MMP9, and VEGFA expression by western blot analysis after inhibiting HK2 expression in the L1‐OE and vector control groups of U87, T98, and GBM1 glioma cell lines (*n* = 3 replicates). One‐way ANOVA followed by Tukey's multiple comparisons test was used to generate *P* values. Data expressed as mean ± SEM. *P < 0.05, **P < 0.01, ***P < 0.001, and ****P < 0.0001. HK2, hexokinase 2; L1, neural cell adhesion molecule L1; L1‐OE, L1‐overexpressing; MMP2, matrix metalloproteinase 2; MMP9, matrix metalloproteinase 9; siHK2‐2, HK2 knockdown; siNC, scramble siRNA control; VC, vector control; VEGFA, vascular endothelial growth factor A.

To obtain further evidence that the miR‐143‐3p‐regulated HK2 is the downstream regulator of L1‐mediated VM formation and tumor invasion in glioma, we next detected HK2 expression in the L1‐overexpressing and control groups of different glioma cell lines with or without miR‐143‐3p mimic transfection. The results demonstrated that HK2 expression was increased in L1‐overexpressing glioma cells. In contrast, treatment with the miR‐143‐3p mimic significantly inhibited HK2 expression regardless of overexpression of L1 in glioma cells (Fig. 6A). Moreover, siRNA‐mediated knockdown of HK2 expression reversed L1‐enhanced tube formation (Fig. 6B) and tumor invasion (Fig. 6C) in different glioma cell lines. Additionally, western blot analysis revealed that inhibition of HK2 expression showed significant effects on L1‐mediated upregulation of VEGFA, MMP2, and MMP9 expression in glioma cells (Fig. 6D,E), suggesting that L1 may promote VM formation and tumor invasion via L1/HK2 cascade‐mediated regulation of VEGFA, MMP2, and MMP9 expression in glioma cells.

Several previous studies demonstrated that the maintenance of cancer stem cells (CSCs) properties is critical for VM formation [10, 47]. To confirm whether the maintenance of glioma stem cells (GSCs) is associated with L1/HK2 cascade‐regulated VM formation, we performed the sphere assay, and examined CD133 expression in combination with L1, miR‐143‐3p, and HK2. The results showed that L1 overexpression results in the upregulation of the expression of CD133, the biomarker of GSCs, in U87, T98, and GBM1 glioma cells (Fig. S4A). In addition, the neurosphere assay revealed that L1 overexpress could enhance the characteristic of GSCs and upregulate CD133 expression in different glioma cell lines (Fig. S4B,C). In contrast, the blockade of the miR‐143‐3p/HK2 cascade would potently disrupt neurosphere formation, and downregulate CD133 expression (Fig. S4B,C), to reverse L1‐enhanced glioma stem cell growth. Thus, these results suggested that L1‐maintained GSCs might play a role in VM formation.

### 3.7 Activation of the PI3K/AKT signaling pathway is involved in L1‐mediated VM formation by glioma cells

**Fig. 7:**
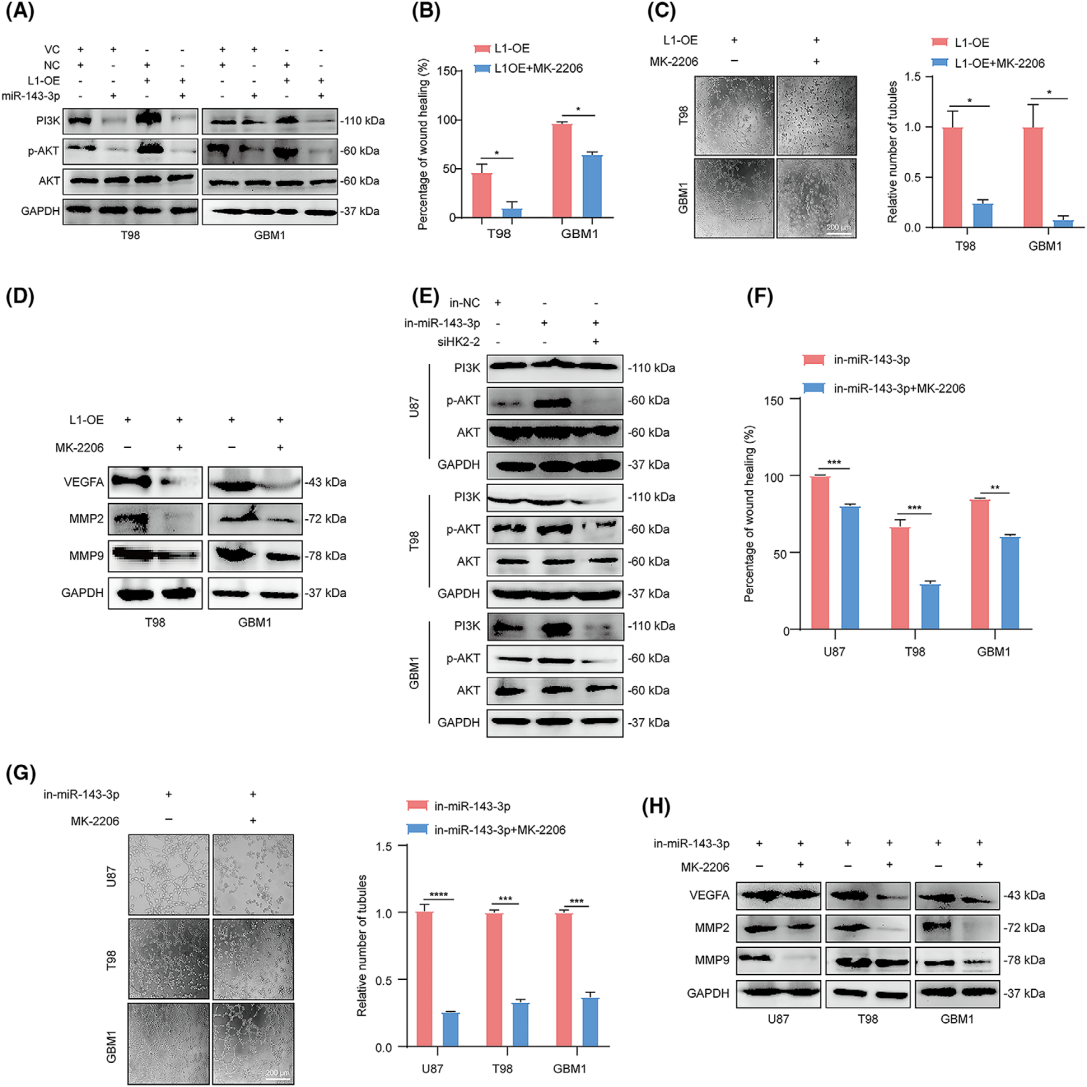
Activation of the PI3K/AKT signaling pathway is essential for L1‐mediated VM formation by glioma cells. (A) Determination of PI3K, p‐AKT, and AKT levels in the miR‐143‐3p mimic‐treated L1‐OE and vector control groups of T98, and GBM1 glioma cell lines by western blot analysis (*n* = 3 replicates). (B) The inactivation of AKT signaling suppresses L1 overexpression‐enhanced glioma invasion in T98 and GBM1 cells (*n* = 3 replicates). (C) Representative images and quantification of tube formation in L1‐overexpressing T98 and GBM1 glioma cells with or without MK‐2206 treatment (*n* = 3 replicates). Scale bar, 200 μm. (D) Western blot analysis of MMP2, MMP9, and VEGFA expression in L1‐overexpressing T98 and GBM1 glioma cells with or without MK‐2206 treatment (*n* = 3 replicates). (E) Determination of PI3K, p‐AKT, and AKT levels in different glioma cell lines by western blot analysis after blocking the L1/HK2 cascade (*n* = 3 replicates). (F,G) MK‐2206 treatment can reverse the enhancement of the tumor invasion (F) and tube formation (G) capabilities induced by in‐miR‐143‐3p transfection in glioma cells (*n* = 3 replicates). Scale bar, 200 μm. (H) The determination of MMP2, MMP9, and VEGFA expression in in‐miR‐143‐3p‐transfected glioma cells with or without MK‐2206 treatment (*n* = 3 replicates). A two‐tailed Student's *t*‐test was used to generate *P* values. Data expressed as mean ± SEM. *P < 0.05, **P < 0.01, ***P < 0.001, and ****P < 0.0001. HK2, hexokinase 2; in‐miR‐143‐3p, miR‐143‐3p inhibitor; in‐NC, miR‐143‐3p inhibitor negative control; L1, neural cell adhesion molecule L1; L1‐OE, L1‐overexpressing; MMP2, matrix metalloproteinase 2; MMP9, matrix metalloproteinase 9; p‐Akt, phosphorylated Akt; siHK2‐2, HK2 knockdown; VC, vector control; VEGFA, vascular endothelial growth factor A.

Since previous studies demonstrated that activation of the PI3K/AKT signaling pathway may play a role in VM formation by glioma cells [20, 53], we then determined the correlation between the PI3K/AKT signaling pathway and L1 expression in glioma cells. The results indicated that the PI3K/AKT signaling pathway was induced by L1 overexpression, whereas treatment with the miR‐143‐3p mimic significantly inactivated PI3K/AKT signaling regardless of L1 overexpression in the T98 and GBM1 glioma cell lines (Fig. 7A). Importantly, treatment with MK2206, a specific inhibitor of the AKT signaling pathway, thoroughly blocked L1‐enhanced tumor invasion and tube formation in glioma cells (Fig. 7B,C). In addition, we found that the expression levels of VEGFA, MMP2, and MMP9 were, unsurprisingly, downregulated after inhibition of AKT signaling, even in L1‐overexpressing glioma cells (Fig. 7D).

To gain further insight into the regulatory mechanism of L1‐mediated VM formation by glioma cells, we attempted to confirm whether the activation of AKT signaling was impaired after inhibition of the L1/HK2 cascade in glioma cells. Western blot analysis demonstrated that the AKT signal was activated after treatment with the miR‐143‐3p inhibitor and inactivated when glioma cells were transfected with HK2 siRNA (Fig. 7E). In addition, after treatment with the MK‐2206 inhibitor, tumor invasion and tube formation were inhibited in different glioma cell lines treated with the miR‐143‐3p inhibitor (Fig. 7F,G). Mechanistically, we then assessed the protein levels of VEGFA, MMP2, and MMP9 in glioma cells after treatment with the miR‐143‐3p inhibitor and/or MEK‐2206. Our results showed that inactivation of AKT signaling markedly decreased the expression levels of VEGFA, MMP2, and MMP9 in the U87, T98, and GBM1 glioma cell lines even after treatment with the miR‐143‐3p inhibitor (Fig. 7H), suggesting that the PI3K/AKT signaling pathway may be the key downstream factor of L1/HK2 cascade‐mediated VM formation process.

### 3.8 L1‐targeted strategy increases the efficacy of antiangiogenic therapy

**Fig. 8:**
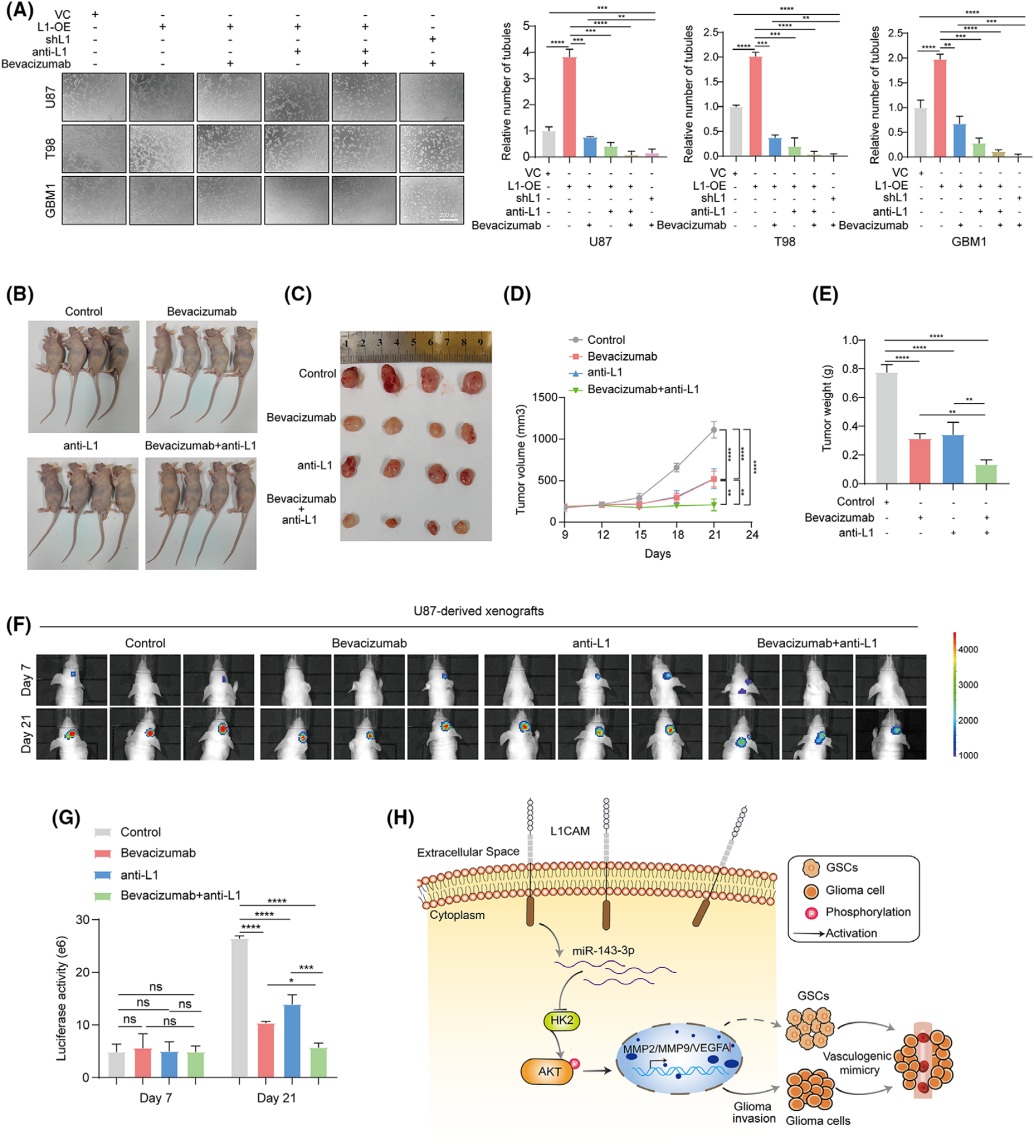
The blockade of L1/HK2 cascade enhance the efficacy of antiangiogenic therapy *in vivo* and *in vitro*. (A) Determination and quantification of tube formation in L1‐overexpression (L1‐OE), L1‐inhibited (shL1), or vector control glioma cell lines treated with bevacizumab and/or anti‐L1 neutralizing monoclonal antibody (anti‐L1; *n* = 3 replicates). Scale bar, 200 μm. (B–D) The tumor growths of bevacizumab and/or anti‐L1 treated different groups (*n* = 4 for each group) in subcutaneous U87‐xenografted BALB/c‐nu mice at the indicated timepoints (9, 12, 15, 18, 21, and 24 days). (E) Tumor weight of bevacizumab and/or anti‐L1 treated different groups (*n* = 4 for each group) in subcutaneous U87‐xenografted BALB/c‐nu mice. (F,G) *In vivo* bioluminescent images (F) and the quantification (G) of intracranial U87‐derived xenografts treated with bevacizumab and/or anti‐L1 at the indicated timepoints (7 and 21 days, *n* = 4 for each group). (H) Schematic of the mechanism of L1‐mediated VM formation in glioma. The broken arrow indicates that the signaling pathway is not yet fully verified. One‐way ANOVA followed by Tukey's multiple comparisons test was used to generate *P* values. Data expressed as mean ± SEM. *P < 0.05, **P < 0.01, ***P < 0.001, and ****P < 0.0001. HK2, hexokinase 2; L1, neural cell adhesion molecule L1; L1‐OE, L1‐overexpressing; MMP2, matrix metalloproteinase 2; MMP9, matrix metalloproteinase 9; ns, not significant; shL1, L1 knockdown; VC, vector control; VEGFA, vascular endothelial growth factor A.

Since L1 overexpression promotes VM formation in glioma cells, our hypothesis is that by treating tumors with the inhibition of L1, drug resistance will be attenuated, and tumor growth rendered more susceptible to inhibiting by an antiangiogenic drug. We therefore applied human glioma cells and xenografted mice to evaluate the potential effects of L1 on antiangiogenic therapy *in vivo* and *in vitro*. Tube angiogenic assay indicated that an antiangiogenic drug: bevacizumab, a VEGF‐specific inhibitor, could enhance its antiangiogenic capability, when used in combination with anti‐L1 neutralizing monoclonal antibody (anti‐L1), or in L1‐inhibited glioma cell lines (shL1; Fig. 8A). In addition, tumors were established with the treatments of anti‐L1 neutralizing monoclonal antibody (anti‐L1) and/or bevacizumab in subcutaneous (*n* = 4) or orthotopically (*n* = 3) xenografted BALB/c‐nu mice. The results indicated that the combination of the anti‐L1 strategy and bevacizumab significantly suppress glioma growth and increase efficacy beyond that of the antiangiogenic drug alone (Fig. 8B–G), suggesting that the L1‐targeted strategy might efficiently improve the effect of antiangiogenic treatment for glioma patients.

## 4 Discussion

The tumor microenvironment refers to the environment in which tumors are located during tumorigenesis, growth, and metastasis and includes tumor cells, immune cells, and endothelial cells, as well as soluble substances such as cytokines and peptide growth factors [54]. In this context, the blood vessels inside the tumor provide nutrients to contribute to tumorigenesis, tumor development, and metastasis. Thus, in the past decade antiangiogenic therapy, which could inhibit endothelial cell proliferation and endothelial cell migration to hypoxic tumor tissue and hinder the supply of oxygen and nutrition to tumor cells, has become an effective cancer treatment strategy [55]. However, more evidence indicates that VM is closely related to resistance to antiangiogenic therapies in various malignancies [56]. In glioma, patients with VM formation have a higher degree of malignancy, higher aggressiveness, and poorer prognosis than those without VM formation [57, 58], suggesting that the clinical strategy for targeting VM may improve the prognosis of glioma patients.

The functional plasticity associated with VM formation was first described in aggressive human uveal melanomas [9]. The potential regulatory mechanism is associated with some critical signaling molecules involved in tumor metastasis, invasion, and matrix reconstruction, such as vascular endothelial cadherin (VE‐cadherin), VEGFA, EphA2, PI3K/AKT signals, MMPs, and HIF‐1α. In this context, VEGFA has been reported to regulate the proliferation, migration, and survival of vascular endothelial cells by activating its receptor VEGFR2 on vascular endothelial cells [59], as well as positively regulating angiogenesis and VM formation [60]. In addition, MMPs, which are known as critical regulators in glioma invasion [61], have been found to be potential biomarkers of VM formation in lung cancer and melanoma [62, 63, 64]. Notably, a recent study indicated that IGFBP2 may act as a stimulator of VM formation by upregulating MMP2 expression in glioma cells [65], indicating that activation of MMPs might be essential for VM formation in glioma.

Previous studies demonstrated that AKT signaling plays major roles in regulating not only cell metabolism, proliferation, and survival [66, 67] but also VM formation. Yu et al. reported that the Chk‐PKM2 axis could promote metabolic control of VM formation in p53‐mutated triple‐negative breast cancer (TNBC) [68]. Another study indicated that Sal‐A could block VM network formation in NSCLC cells by modulating the PI3K/AKT/mTOR signaling pathway [69]. In addition, in human prostate cancer cells investigators revealed that epigallocatechin‐3‐gallate (EGCG) results in the inhibition of VM by suppressing the Twist/VE‐cadherin/AKT pathways in human PC‐3 cells [70]. Notably, Liang et al. [64] found that the suppression of Rictor expression could affect VM formation by regulating AKT activation and MMP‐2/9 expression in melanoma, suggesting that AKT activation‐involved MMP‐2/9 regulation might be a potential regulatory mechanism of VM formation.

Multiple miRNAs have been reported to be associated with the regulation of tumorigenesis, tumor development, invasion and metastasis, and angiogenesis in human cancers [71, 72, 73]. In this context, we identified miR‐143‐3p, which is significantly regulated by L1 overexpression in glioma cells (Fig. 4B) and may play a role in L1‐mediated invasion and VM formation in glioma (Fig. 4C–F). Previous studies have indicated that miR‐143‐3p is involved in tumor proliferation, metastasis, and invasion by regulating different tumor‐related factors in various cancers. Wang et al. [74] showed that miR‐143‐3p could promote brain metastasis of lung cancer. In hepatocellular carcinoma cells, miR‐143‐3p was found to inhibit proliferation and invasion by regulating its target gene, *FGF1* [75]. In addition, more evidence has shown that miR‐143‐3p is essential for proliferation, migration, and invasion in osteosarcoma and laryngeal squamous cell carcinoma [76, 77]. A previous report showed that miR‐143‐3p modulates both cellular and exosome‐mediated responses, thereby regulating the crosstalk of endothelial cells and smooth muscle in pulmonary arterial hypertension [78]. Importantly, a very recent study showed that miR‐143‐3p targeting of *ITGA6* was involved in tumor growth and angiogenesis by downregulating PLDF expression in gallbladder carcinoma [79]. Notably, we also found that the regulation of miR‐143‐3p targeting of *HK2* could affect tumor growth and the Warburg effect in breast cancer [80], which is similar to our results that miR‐143‐3p directly targets the *HK2*‐3'UTR binding site in glioma cells (Fig. 5F).

Accumulated reports have demonstrated that the maintenance of cancer stem cell (CSC) properties is critical for VM formation [10, 47, 81]. Several studies revealed that the properties of breast cancer stem cells contribute to VM. Izawa et al. [82] indicated that breast cancer stem cells from TNBC are associated with the initiation of VM formation in Matrigel. In addition, overexpression of stemness‐related OCT4 can enhance CSC properties and VM formation, which subsequently promotes intravasation of breast cancer cells [83]. The regulation of epithelial‐mesenchymal transition (EMT) and cancer stem cell properties were found to be closely linked to ZEB1‐mediated VM formation in prostate cancer cells [84]. Intriguingly, previous investigations showed that L1 is required for maintaining the growth and survival of GSCs [85], suggesting that L1‐mediated maintenance of GSCs might be involved in VM formation by glioma cells. Moreover, evidence has revealed that miR‐143 could inhibit the properties of CSCs in breast cancer and prostate cancer [86, 87]. In retinoblastoma tumors, stemness‐related SOX2 is usually accompanied by higher VEGFA expression and tumor invasion [88]. Thus, these findings imply that L1‐mediated maintenance of GSCs might also be associated with VM formation through miR‐143‐3p‐regulated HK2‐mediated activation of the PI3K/AKT signaling pathway in glioma cells.

## 5 Conclusion

In summary, as shown in the diagram in Fig. 8H, we clarified the potential mechanism of L1‐enhanced VM formation in glioma. Our results indicated that L1 overexpression positively correlates with glioma invasion and VM formation, as well as the pathological grades and prognosis in glioma. In addition, we further identified that the L1‐regulated miR‐143‐3p and HK2 was involved in this process by regulating MMP2/9 and VEGFA expression via modulation of the PI3K/AKT signaling pathway. In addition, L1‐mediated the maintenance of GSCs might also be involved in VM formation in glioma cells. In xenografted mice, the results indicated that the inhibition of L1 expression significantly diminish the antiangiogenic therapy resistance. Together, our findings uncovered a new function of L1 in VM formation and tumor invasion in glioma, which may partially explain the reason for the positive correlation between high L1 expression and poor prognosis in glioma patients [89]. Therefore, this L1/HK2 cascade can be considered a potential clinical target for reducing VM formation to improve the therapeutic benefit of antiangiogenic treatment for patients with glioma.

## Conflict of interest

The authors declare no conflict of interest.

## Author contributions

YH and CZ contributed to the collection and assembly of data, data analysis and interpretation, and methodology. PL, FO, JL, and CL contributed data analysis and interpretation. BT and XY supervised, obtained funding, wrote & revised the article. All authors approved the article for publication.

## Supporting information


**Fig. S1.** The qPCR confirmation of miRNA‐seq identified top 10 regulated miRNAs in glioma cells.

**Fig. S2.** Survival curves of patients with glioma presenting about BRD2 or SECISBP2L in the GEPIA database.

**Fig. S3.** The blockade of L1/HK2 cascade significantly suppresses the capabilities of tumor invasion and tube formation in glioma cells.

**Fig. S4.** The regulation of L1/HK2 cascade is involved in the maintenance of GSCs.

**Table S1.** List of primer sequences for qRT‐PCR of miRNAs and genes.
**Table S2.** The sources of the primary antibodies.
**Table S3.** Correlation between L1 expression and clinicopathologic characteristics of glioma specimens.
**Table S4.** GO enrichment analysis of differentially expressed genes (shL1 vs. VC control).

**Data S1.** Figure legends.

## Data Availability

The miRNA‐seq data and RNA‐seq data have been deposited in the Sequence Read Archive of the National Center for Biotechnology Information under accession numbers PRJNA820468 and PRJNA851207, respectively.
