# Supplementary figures and images for: L1CAM promotes vasculogenic mimicry formation by miR‐143‐3p‐induced expression of hexokinase 2 in glioma
Source: Mol Oncol. 2023 Feb 8;17(4):664–85. doi: 10.1002/1878-0261.13384 (PMC10061292; doi:10.1002/1878-0261.13384)

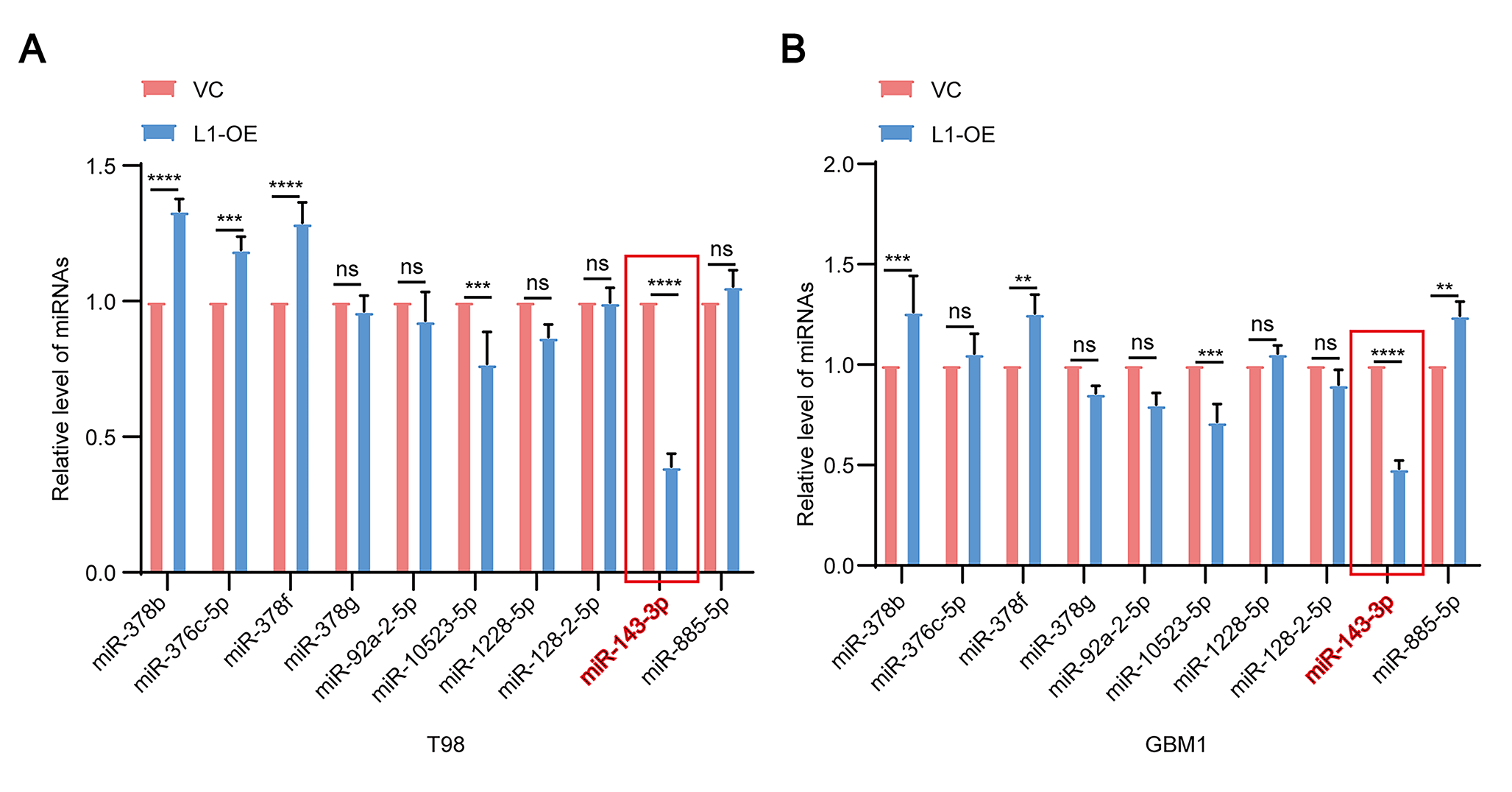

Supplement: Supplementary file 1 — Fig. S1. The qPCR confirmation of miRNA‐seq identified top 10 regulated miRNAs in glioma cells. [file MOL2-17-664-s005.tif]

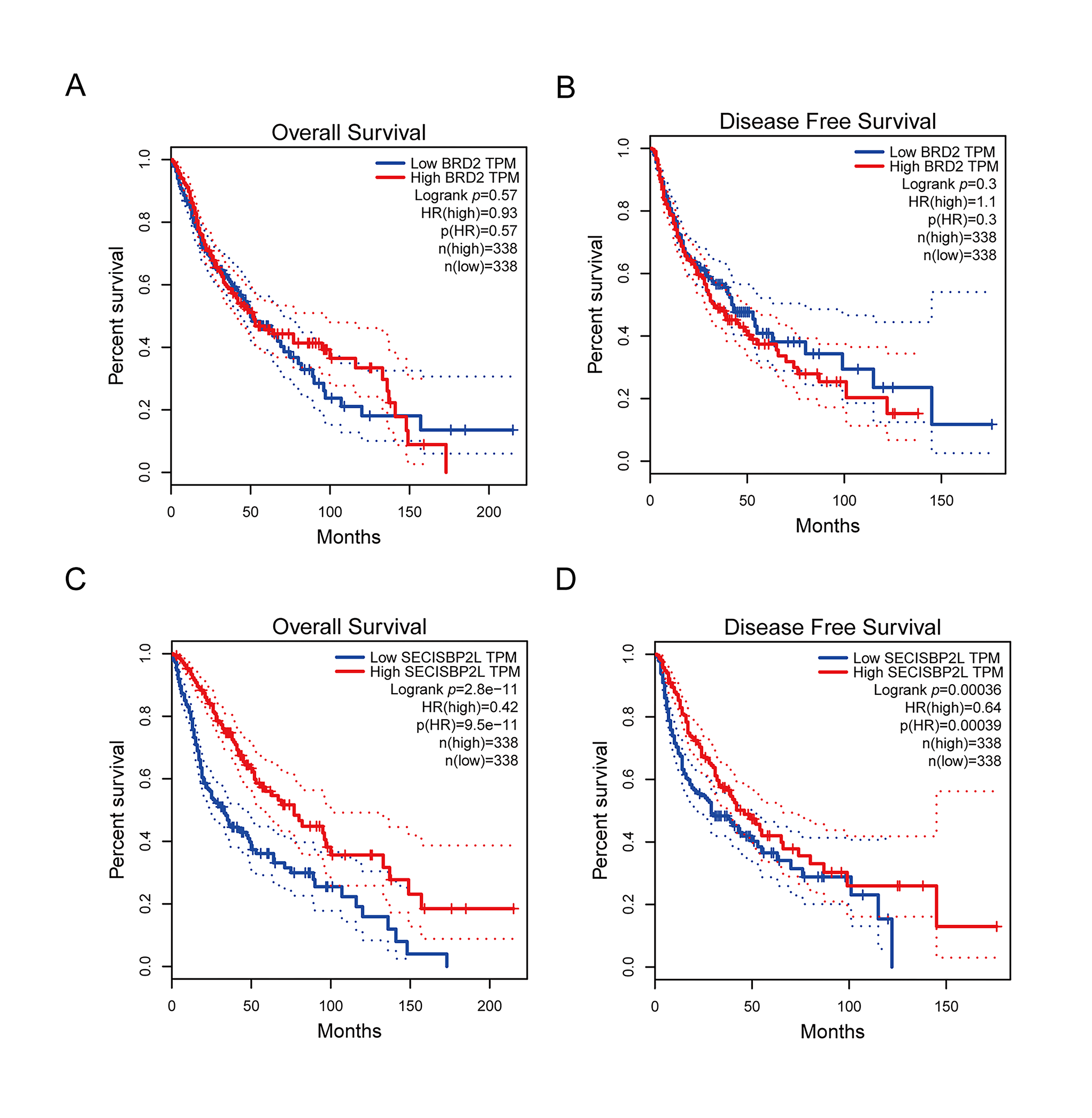

Supplement: Supplementary file 2 — Fig. S2. Survival curves of patients with glioma presenting about BRD2 or SECISBP2L in the GEPIA database. [file MOL2-17-664-s003.tif]

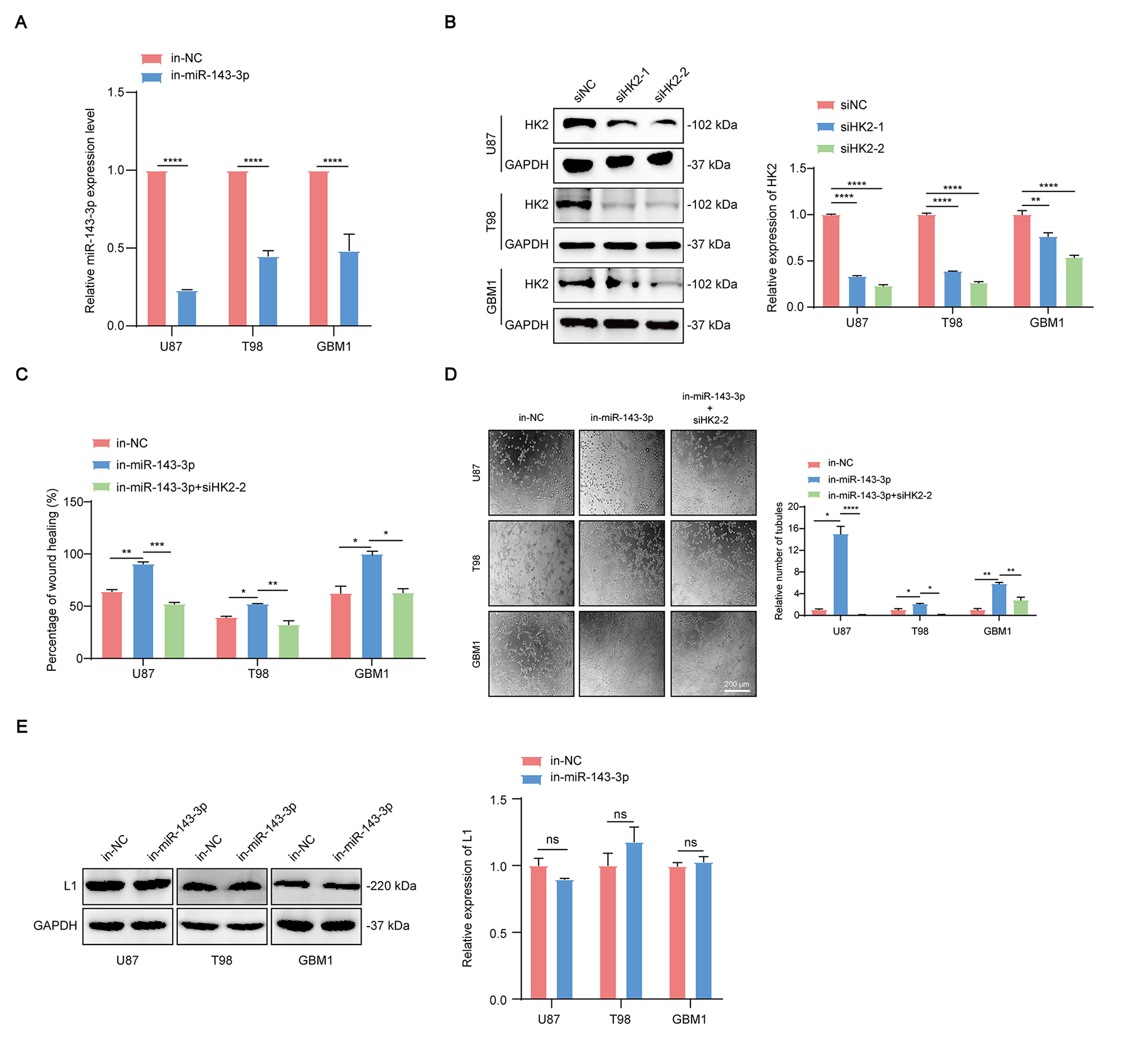

Supplement: Supplementary file 3 — Fig. S3. The blockade of L1/HK2 cascade significantly suppresses the capabilities of tumor invasion and tube formation in glioma cells. [file MOL2-17-664-s001.tif]

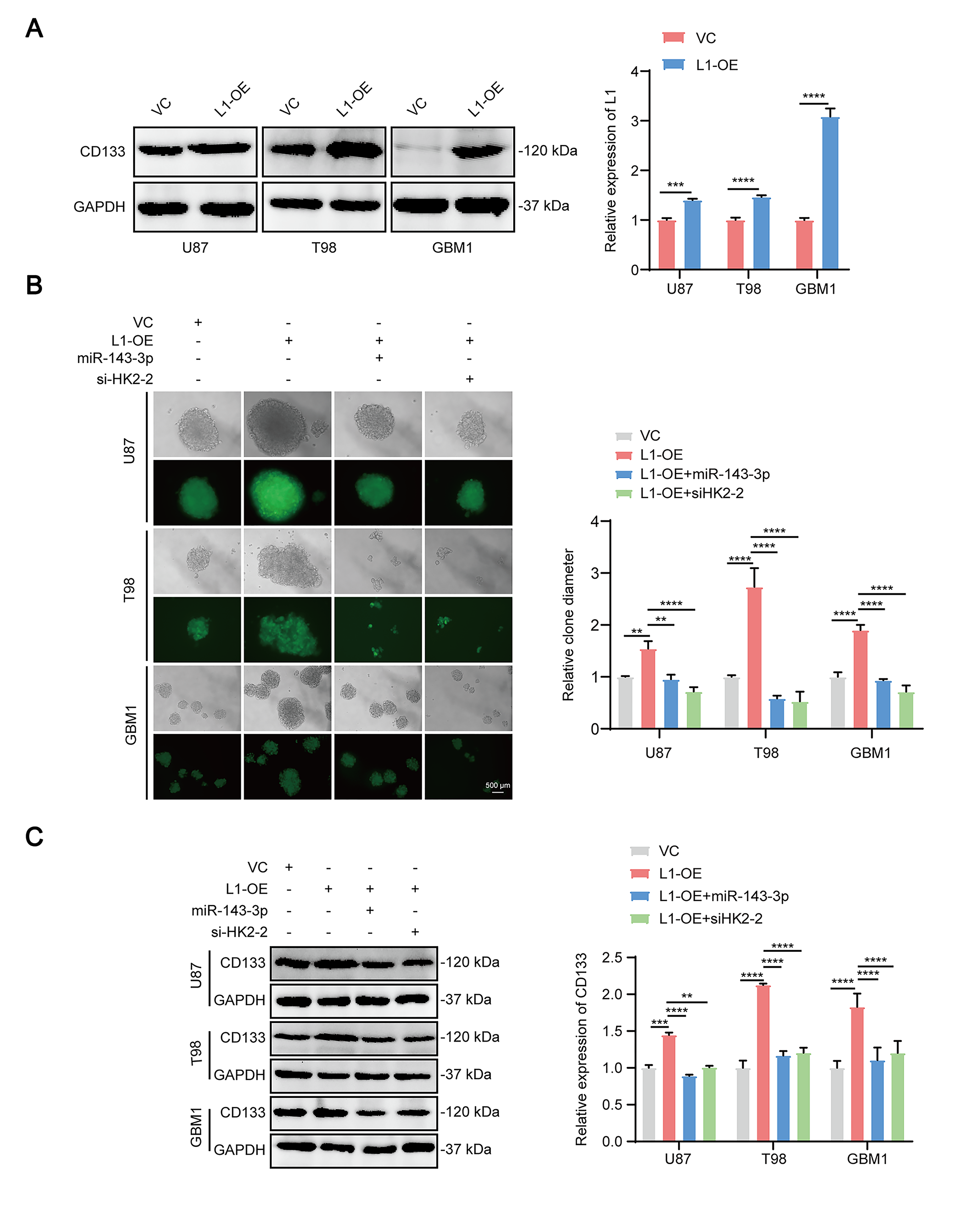

Supplement: Supplementary file 4 — Fig. S4. The regulation of L1/HK2 cascade is involved in the maintenance of GSCs. [file MOL2-17-664-s004.tif]
