# Supplementary material for: L1CAM promotes vasculogenic mimicry formation by miR‐143‐3p‐induced expression of hexokinase 2 in glioma
Source: Mol Oncol. 2023 Feb 8;17(4):664–85. doi: 10.1002/1878-0261.13384 (PMC10061292; doi:10.1002/1878-0261.13384)
Supplement: Supplementary file 5 — Table S1. List of primer sequences for qRT‐PCR of miRNAs and genes. Table S2. The sources of the primary antibodies. Table S3. Correlation between L1 expression and clinicopathologic characteristics of glioma specimens. Table S4. GO enrichment analysis of differentially expressed genes (shL1 vs. VC control). [file MOL2-17-664-s006.docx]

**Table S1.** List of primer sequences for qRT-PCR of miRNAs and genes

| Primer Name | Sequence (5′to 3′) |
| --- | --- |
| *hsa-miR-378f* | F: CCTGTGCAACTGGACTTGGAG |
| *hsa-miR-376c-5p* | F: AAGCGCCTGGTGGATATTCCT |
| *hsa-miR-378b* | F: AACAATACTGGACTTGGAGGCA |
| *hsa-miR-378g* | F: AAGTTGCAACTGGGCTTGGAG |
| *hsa-miR-92a-2-5p* | F: AATGCTTGGGTGGGGATTTGTT |
| *hsa-miR-10523-5p* | F: AATGCACGACAATGATGAGAAGAC |
| *hsa-miR-1228-5p* | F: TAATAAGTGGGCGGGGGCAG |
| *hsa-miR-128-2-5p* | F: AACTTGATGGGGGCCGATACA |
| *hsa-miR-143-3p* | F: CTCGACCGTGAGATGAAGCAC |
| *hsa-miR-885-5p* | F: ACATTGGCTCCATTACACTACCC |
| *miR* | R: mRQ 3’ primer supplied from the kit |
| *U6* | F: GGAACGATACAGAGAAGATTAGC |
|  | R: TGGAACGCTTCACGAATTTGCG |
| *KRAS* | F: GAGTACAGTGCAATGAGGGAC |
|  | R: CCTGAGCCTGTTTTGTGTCTAC |
| *BRD2* | F: GGAAACATCAGTTCGCATGGC |
|  | R: CACTCTGAAGCAGCCCAATAA |
| *HK2* | F: GAGCCACCACTCACCCTACT |
|  | R: CCAGGCATTCGGCAATGTG |
| *SECISBP2L* | F: TATACGATGGCAACAACCCAATC |
|  | R: AGAAACCCATAACCTGGGCAC |
| *GAPDH* | F: GTGATGGGTGTGAACCACGA |
|  | R: CACAGTCTTCTGGGTGGCAG |

**Table S2.** The sources of the primary antibodies

| Antibodies | Suppliers | Catalog Number | Concentration | Experiments |
| --- | --- | --- | --- | --- |
| L1CAM | BioLegend | 838101 | 1:2000 / 1:200 | Western blot / IHC |
| GAPDH | Boster | BM1623 | 1:5000 | Western blot |
| Hexokinase 2 | Proteintech | 22029-1-AP | 1:2000 / 1:200 | Western blot / IHC |
| MMP2 | Proteintech | 66366-1-Ig | 1:2000 / 1:150 | Western blot / IHC |
| MMP9 | Proteintech | 10375-2-AP | 1:2000 / 1:150 | Western blot / IHC |
| VEGFA | Proteintech | 66828-1-Ig | 1:2000 | Western blot |
| N-cadherin | Proteintech | 66219-1-Ig | 1:2000 | Western blot |
| Vimentin | Proteintech | 60330-1-Ig | 1:2000 | Western blot |
| E-cadherin | Proteintech | 20874-1-AP | 1:5000 | Western blot |
| CD133 | Proteintech | 18470-1-ap | 1:2000 | Western blot |
| EPHA2 | ABclonal | A7183 | 1:2000 | Western blot |
| PI3K | Proteintech | 20584-1-AP | 1:2000 | Western blot |
| p-AKT | Cell Signaling | #4060 | 1:2000 | Western blot |
| AKT | Cell Signaling | #4691 | 1:2000 | Western blot |
| CD31 | Boster | BM4213 | 1:2000 / 1:100 / 1:100 | Western blot / IHC / IF |
| CD34 | Proteintech | 14486-1-Ig | 1:1000 / 1:300 / 1:100 | Western blot / IHC / IF |
| L1CAM | InVivo BioTech | UJ127.11 | 300 μg/mouse | Drug treatment |

**Table S3.** Correlation between L1 expression and clinicopathologic characteristics of glioma specimens

| Characteristics | L1 expression | | *P* value |
| --- | --- | --- | --- |
|  | Low | High |  |
| Age (years) |  |  | 0.401 |
| ≤ 60 | 80 (44.4) | 75 (41.7) |  |
| > 60 | 10 (5.6) | 14 (7.8) |  |
| N/A | 0 (0) | 1 (0.5) |  |
| Gender |  |  | 0.065 |
| Male | 50 (27.8) | 62 (34.4) |  |
| Female | 40 (22.2) | 28 (15.6) |  |
| Tumor grades |  |  | 0.001 |
| I + II | 63 (35) | 42 (23.3) |  |
| III + IV | 27 (15) | 48 (26.7) |  |
| Recurrence, n (%) |  |  | 0.003 |
| Yes | 38 (21.1) | 58 (32.2) |  |
| No | 52 (28.9) | 32 (17.8) |  |

N/A, not available

**Table S4.** GO enrichment analysis of differentially expressed genes (shL1 vs. VC control)

| **Description** | ***P*-value** | **geneID** |
| --- | --- | --- |
| **Cell motility** | 2.67E-08 | AGT/BDKRB1/C5AR1/CD74/EDN1/F2RL1/F3/F10/FGF10/FUT1/ONECUT1/ICAM1/IGFBP5/ITGAX/ITGB3/LAMC2/MMP2/MMP9/PDGFB/PTGS2/RARRES2/TACR2/TEK/TNF/VEGFA/TNFRSF14/GPNMB/DAPK2/SEMA5B/SEMA3G/ACKR3/TCAF2 |
| **Cell migration** | 3.51E-08 | AGT/BDKRB1/C5AR1/CD74/EDN1/F2RL1/F3/F10/FGF10/FUT1/ONECUT1/ICAM1/IGFBP5/ITGAX/ITGB3/LAMC2/MMP2/MMP9/PDGFB/PTGS2/RARRES2/TEK/TNF/VEGFA/TNFRSF14/GPNMB/DAPK2/SEMA5B/SEMA3G/ACKR3/TCAF2 |
| **Cell adhesion** | 2.48E-09 | ANK3/RUNX3/CD74/DPP4/EGR3/ELANE/FBLN2/FUT1/GLI2/HLA-DMB/HLA-DPA1/IL12RB1/ITGB2/PCK1/PDGFB/SAA1/TEK/TGM2/TNF/VEGFA/NCK2/TNFRSF14/DNAJA3/PTPRU/EBI3/SIRPB1/CD160/NOD2/NDNF/EMILIN2/ABAT/CEACAM1/DUSP1/ONECUT1/MMP2/LAPTM5/GPNMB/AJAP1/CAMSAP3/CD33/CHRNB2/F2RL1/FGF10/INHA/RORA/TSPAN32/SLAMF8/CRLF2/TTBK1/CYGB/CSF2 |
| **Inflammatory response** | 1.93E-09 | SERPINA3/ADM/BDKRB1/BDKRB2/C3/C5AR1/CHI3L1/ELANE/F2RL1/F3/HP/ITGB2/KRT16/MAPT/NFATC4/SERPINA1/PTGS1/PTGS2/RARRES2/SAA1/TFR2/TNF/SCG2/GPR68/CHST1/AOC3/MGLL/IL36RN/TREM1/SLAMF8/FFAR4/PLA2G4B |
| **Angiogenesis** | 5.24E-09 | CEACAM1/COL15A1/EDN1/EGR3/EPHB1/FAP/FGF10/GBX2/ITGB3/MMP2/NFATC4/PTGS2/RORA/TEK/VEGFA/SCG2/APLN/SH2D2A/RAMP1/CD160/ANGPTL4/ACKR3/NDNF/UNC5B |
| **Tube morphogenesis** | 1.74E-12 | ADM/CEACAM1/COL15A1/EDA/EDN1/EGR3/EPHB1/ESR1/FAP/FGF10/GBX2/GLI2/HOXA13/ITGB3/LOX/MMP2/NFATC4/NTRK2/PAX2/PTGS2/RORA/TEK/TGM2/VEGFA/WNT6/SCG2/DCHS1/APLN/SH2D2A/NOG/CELSR1/RAMP1/CD160/COBL/ANGPTL4/ASB2/ACKR3/SALL4/VANGL2/NDNF/DNAAF1/UNC5B |
| **Blood vessel morphogenesis** | 7.72E-10 | ADM/CEACAM1/COL15A1/EDN1/EGR3/EPHB1/FAP/FGF10/GBX2/HOXA13/ITGB3/LOX/MMP2/NFATC4/NTRK2/PTGS2/RORA/TEK/VEGFA/SCG2/APLN/SH2D2A/NOG/RAMP1/CD160/ANGPTL4/ACKR3/NDNF/UNC5B |
| **Blood vessel development** | 8.2E-10 | ADM/CEACAM1/COL15A1/DLX3/EDN1/EGR2/EGR3/EPHB1/FAP/FGF10/GBX2/HOXA13/ITGB3/LOX/MMP2/NFATC4/NTRK2/PDGFB/PTGS2/RORA/TEK/VEGFA/SCG2/APLN/SH2D2A/NOG/RAMP1/CD160/ANGPTL4/ACKR3/NDNF/UNC5B |
| **Neuron projection guidance** | 1.06E-09 | CRMP1/EDN1/EFNA2/EGR2/EPHB1/EVX1/GBX1/GBX2/GLI2/L1CAM/LAMC2/VEGFA/KALRN/NOG/DPYSL4/NFASC/SEMA5B/SEMA3G/IGSF9/UNC5B/DRAXIN |
| **Chemotaxis** | 5.7E-13 | C5AR1/CRMP1/DPP4/EDN1/EFNA2/EGR2/EGR3/EPHB1/EVX1/FGF10/GBX1/GBX2/GLI2/HOXB9/ITGB2/ITGB3/L1CAM/LAMC2/LOX/PDGFB/RARRES2/SAA1/VEGFA/SCG2/KALRN/NOG/GPNMB/DPYSL4/NFASC/TREM1/SEMA5B/SLAMF8/SEMA3G/ACKR3/IGSF9/UNC5B/DRAXIN/ACTN2/ANK3/CHRNB2/KRT19/NEB/PAX2/S100B/TTN/GAS7/KLHL41/NEBL/TPPP/COBL/NRN1/MYH14/OBSCN/SLITRK6/ADPRHL1/MAPT/MMP2/RTN4RL2/COL15A1/HOXA13/ITPR1/TEK/DCHS1/LAMC3/SRCIN1/ADM/NCK2/CAMSAP3/ATCAY |
