# Supplementary material for: L1CAM promotes vasculogenic mimicry formation by miR‐143‐3p‐induced expression of hexokinase 2 in glioma
Source: Mol Oncol. 2023 Feb 8;17(4):664–85. doi: 10.1002/1878-0261.13384 (PMC10061292; doi:10.1002/1878-0261.13384)
Supplement: Supplementary file 6 — Data S1. Figure legends. [file MOL2-17-664-s002.docx]

**Supplementary Figure Legends**

**Fig. S1.** The qPCR confirmation of miRNA-seq identified top 10 regulated miRNAs in glioma cells. (A, B) The qPCR confirmation of miRNA-seq identified top 10 regulated miRNAs in L1-OE and VC groups in T98 (A) and GBM1 (B) glioma cells (n = 3 replicates). A two-tailed Student’s *t*-test was used to generate *P* values. Data expressed as mean ± SEM. ***P* < 0.01, ****P* < 0.001, and *****P* < 0.0001. ns, not significant. L1-OE, L1-overexpressing; VC, vector control.

**Fig. S2.** Survival curves of patients with glioma presenting about BRD2 or SECISBP2L in the GEPIA database. (A-D) The overall survival and disease-free survival rates are negatively correlated with BRD2 (A,B) or SECISBP2L (C,D) expression in the glioma patients from the GEPIA database (n = 338). The differences in patients’ survival curves between different subgroups were evaluated by Kaplan-Meier analyses and the statistical significance of differences between the survival curves was assessed with a log-rank test.

**Fig. S3.** The blockade of L1/HK2 cascade significantly suppresses the capabilities of tumor invasion and tube formation in glioma cells. (A) The inhibition of miR-143-3p by transfecting in-NC or in-miR-143-3p in U87, T98, and GBM1 glioma cell lines (n = 3 replicates). (B) The expression levels of HK2 expression in siNC-, siHK2-1-, and siHK2-2-treated U87, T98, and GBM1 glioma cell lines (n = 3 replicates). (C, D) The capabilities of tumor invasion (C) and tube formation (D) were enhanced after transfecting in-miR-143-3p and reversed by inhibiting HK2 expression in different glioma cell lines (n = 3 replicates). (E) Determination and quantification of L1 expression in miR-143-3p-perturbed glioma cells (n = 3 replicates). In (A,B,E), A two-tailed Student’s *t*-test was used to generate *P* values. In (C,D), one-way ANOVA followed by Tukey’s multiple comparisons test was used to generate *P* values. Data expressed as mean ± SEM. **P* < 0.05, ***P* < 0.01, ****P* < 0.001, and *****P* < 0.0001. ns, not significant. L1, neural cell adhesion molecule L1; HK2, hexokinase 2; in-NC, miR-143-3p inhibitor negative control; in-miR-143-3p, miR-143-3p inhibitor; siNC, scramble siRNA control; siHK2-1 and siHK2-2, HK2 knockdown.

**Fig. S4.** The regulation of L1/HK2 cascade is involved in the maintenance of GSCs. (A) The determination and quantification of CD133 expression in L1-OE and VC groups in different glioma cell lines (n = 3 replicates). (B) The neurosphere assay of L1-overexpressing glioma cells transfected with miR-143-3p mimic or HK2 siRNA (n = 3 replicates). Scale bar, Scale bar, 500 μm. (C) The determination and quantification of CD133 expression in L1-overexpressing glioma cells transfected with miR-143-3p mimic or HK2 siRNA (n = 3 replicates). In (A), a two-tailed Student’s *t-*test was used to generate *P* values. In (B,C), one-way ANOVA followed by Tukey’s multiple comparisons test was used to generate *P* values. Data expressed as mean ± SEM. ***P* < 0.01, ****P* < 0.001, and *****P* < 0.0001. L1, neural cell adhesion molecule L1; HK2, hexokinase 2; GSCs, glioma stem cells; L1-OE, L1-overexpressing; VC, vector control; miR-143-3p, miR-143-3p mimic; siHK2-2, HK2 knockdown.
